# High rates of polygyny do not lock large proportions of men out of the marriage market

**DOI:** 10.1073/pnas.2508091122

**Published:** 2025-10-03

**Authors:** Hampton Gaddy, Rebecca Sear, Laura Fortunato

**Affiliations:** ^a^Department of Economic History, London School of Economics, London WC2A 3LZ, United Kingdom; ^b^Leverhulme Centre for Demographic Science, University of Oxford, Oxford OX1 1JD, United Kingdom; ^c^Centre for Culture and Evolution, Brunel University, London UB8 3PH, United Kingdom; ^d^Institute of Human Sciences, University of Oxford, Oxford OX2 6QS, United Kingdom; ^e^Santa Fe Institute, Santa Fe, NM 87501

**Keywords:** demography, polygyny, conflict, political science, cultural evolution

## Abstract

There is a widespread belief, in both the scholarly literature and the popular press, that polygyny prevents large numbers of men from marrying by skewing the sex ratio of the marriage market. In turn, the exclusion of men from marriage is thought to lead to negative outcomes, e.g., by fueling crime and armed conflict. In this paper, we investigate systematically the relationship between polygyny and men’s marriage prospects. First, using a demographic model, we show that marriage markets are skewed sufficiently feminine, under a range of realistic demographic scenarios, to sustain some level of polygyny without locking any men out of marriage. Second, through analysis of 84.1 million census records from 30 countries across Africa, Asia, and Oceania between 1969 and 2016, we show that the subnational association between the prevalence of polygyny and the prevalence of unmarried men is negative or null, rather than positive, for almost all countries in the sample. Third, through analysis of the full-count 1880 US federal census, we show that the average prevalence of unmarried men is lower, not higher, across counties of the West with Mormon polygyny, compared to other counties of the West, and to counties of the Midwest and the Northeast; it is higher only compared to counties of the South. Overall, these findings challenge a dominant narrative linking polygyny to negative social outcomes. Drawing on existing evidence, we suggest that the observed patterns may be explained by an underlying association between the prevalence of polygyny and the strength of promarriage norms.

## Significance

Social scientists often assume that when men can marry multiple wives (polygyny), many other men will be unable to marry. Versions of this assumption feature prominently in theories of civil war, the evolution of monogamy, and the incel movement. Using census data from 30 countries across Africa, Asia, and Oceania, as well as data from the historical United States, we find no clear evidence that polygyny is associated with higher proportions of unmarried men in society. Instead, high-polygyny populations often have marriage markets skewed in favor of men, and actually, men in high-polygyny populations usually marry more than men in low-polygyny ones. These findings challenge entrenched assumptions and inform debates on marriage systems, societal stability, and human rights.

Polygyny—defined as a heterosexual marriage system in which men are allowed to be married to multiple women concurrently—is widespread across societies. In a dataset that is broadly representative of the human cultural variation documented in the ethnographic record (1), 153 of 186 societies (82%) are classified as polygynous, 31 (17%) as monogamous (i.e., any individual may be married to only one spouse at a time), and 2 (1%) as polyandrous (i.e., women are allowed multiple concurrent husbands) (2). The prevalence of polygyny has declined substantially across sub-Saharan Africa and Muslim-majority countries in recent decades, but the practice is still common in both parts of the world, and especially in sub-Saharan Africa (3–5). In the Gambia, for example, data collected in 2019 show that over 45% of children under age 5 resided in a polygynous household, and over 20% of women of reproductive age were in a polygynous union; recent data from countries such as Benin, Chad, Guinea, Mali, Niger, and Senegal show similarly high figures (3). Moreover, there are communities, in both sub-Saharan Africa and elsewhere, in which the practice has recently become more common or taken on new forms (6, 7).

Despite being an important demographic feature of the human past and present, polygyny remains controversial. For example, it is commonly framed as a “harmful cultural practice” by development agencies (8), typically drawing on its posited links to various negative social outcomes like child mortality and intimate partner violence (9, 10). A growing body of work contends that the statistical patterns underpinning those links may be explained away by confounding factors (11–13). One key line of reasoning linking polygyny to negative outcomes has not received the same level of scrutiny, however. Specifically, it is argued that if some men engage in polygyny, other men—“often a majority” (14, p. 12)—will be permanently squeezed out of the marriage market. Implicitly or explicitly, this line of reasoning rests on the assumption that the sex ratio between individuals of marriageable age is equal. For example, an influential thought experiment presented in (15) relies on this assumption to argue that if 25% of men in a population marry 2.6 women on average, then 40% of all men in the population will have to go unmarried. A simplified version of this thought experiment is the widespread notion that if one man marries two women, another man will go unmarried.

Since populations with large proportions of unmarried men are thought to be socially unstable (e.g., 16), many scholars use this line of reasoning to draw a connection between polygyny and negative outcomes at the population level. Some have claimed that polygyny causes high crime rates and violence against women in particular (15, 17, 18). Others have additionally claimed a link to authoritarianism, capital punishment, and discrimination against women in property rights and in access to education (18), and to national military spending (19). Above all, a large body of work in political science has promoted the notion that polygyny is a major determinant of armed conflict, especially civil war (20–25). Over time, these ideas have gained traction in legal scholarship about polygyny (19, 26), in evolutionary research about marriage systems (15, 27, 28), and in popular science books about human nature (29–31). Unsurprisingly, they are frequently picked up in turn by the popular press more broadly [e.g., three articles published in *The Economist* between 2016 and 2021 (32–34)].

Yet the scholarly literature on the topic presents substantial empirical limitations, such as potential reverse causality, omitted variable bias, invalid instrumental variables, and the imprecise measurement of polygyny (*SI Appendix*, section S1). Furthermore, the underlying logic is quite simplistic in its demographic assumptions. For example, the thought experiment in (15) is highly sensitive to the assumption of an equal sex ratio at marriage: Modest deviations from this assumption lead to qualitatively different conclusions. Take, for instance, a population comprising 40 prospective grooms and 50 prospective brides: If 10 of the men (25%) marry two wives each, the remaining 30 men (75%) can still pair up with the remaining 30 women. With 40 prospective grooms and 60 prospective brides, 10% of the men can marry up to six wives each without any of the other men going unmarried.

Through a combination of theoretical modeling and empirical analysis, we provide a systematic investigation of whether polygyny and relatively large numbers of unmarried men do indeed co-occur. We first present a demographic model that details why the “if one man marries two women, another man must go unmarried” view of polygyny is overly simplistic. Then, we use census microdata from 30 countries between 1969 and 2016 to test whether, at the subnational level, a higher prevalence of the practice actually correlates with larger numbers of unmarried men. Finally, we use data from the 1880 census of the United States to investigate the relationship between the presence of Mormon polygyny and the proportion of unmarried men at the county level.

## 1. A Model of Polygynous Marriage Markets

Demographic modeling can be used to assess the effect of polygyny on the sex ratio of marriage markets, including the theoretical limits on men’s chances of marrying heterosexually. Some existing models focus on only a subset of the key variables, such as the female mortality advantage alone (35), the female mortality advantage and the age gap between men and women at marriage (36), or the age gap at marriage and the phenomenon of widow remarriage (37, 38). Other models rely on unrealistic assumptions, such as mortality rates being constant with age (39) or equal between men and women (40). Building on this body of work, we provide a more detailed analysis of how polygyny affects whether all men in a population can secure at least one wife (see *Methods*). Specifically, we model the effect of polygyny on men’s marriage prospects in a demographically stable population that is closed to migration, as a function of five key demographic variables: the life expectancy at birth of men and women, men’s age, the age gap between spouses, and the population growth rate.

**Fig. 1.**
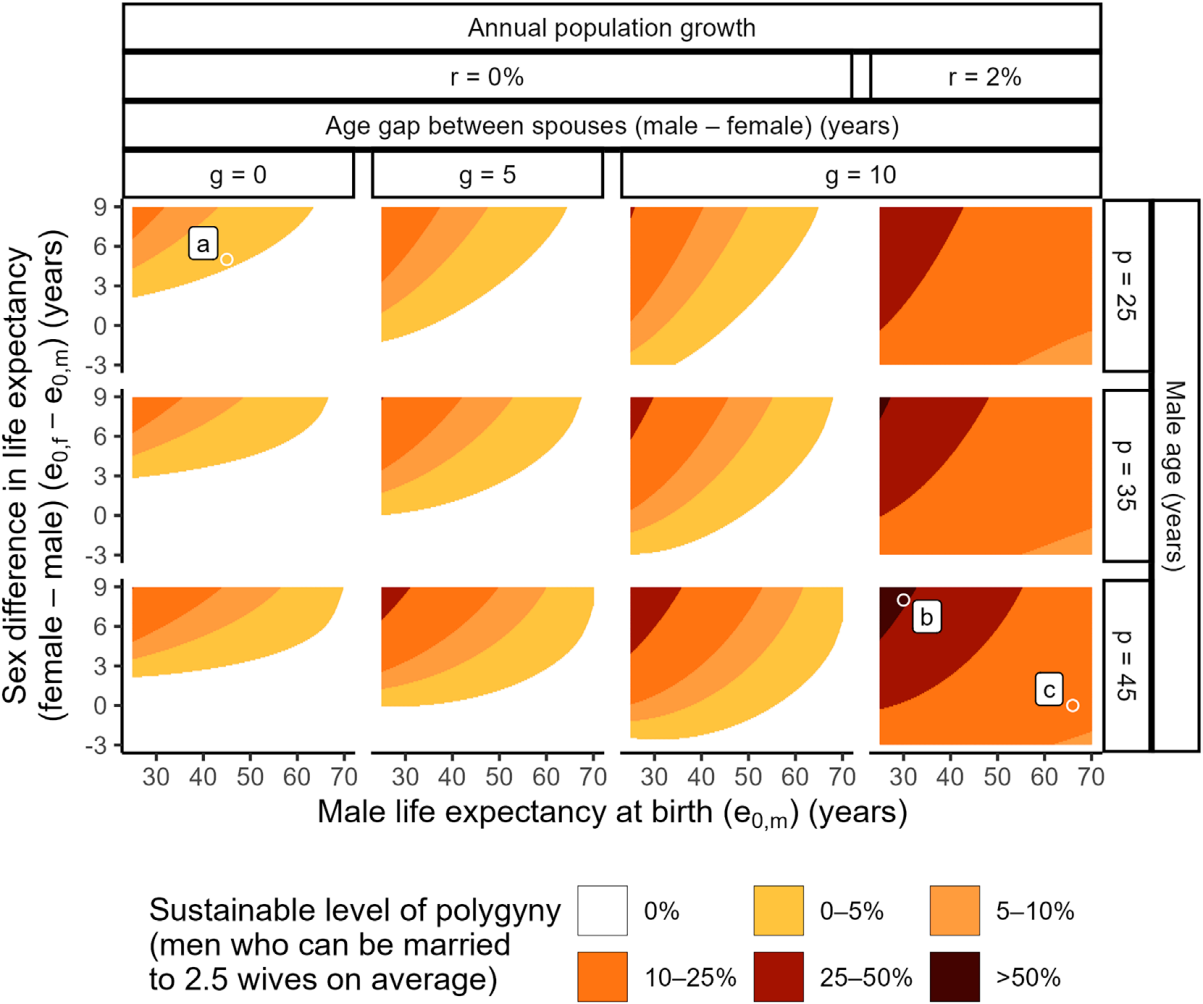
Modeled results of the maximum proportion of men who can be married to 2.5 wives on average, such that all other men of the same age can marry one wife, under a stable population regime that is closed to migration, as a function of female (e_0,f_) and male (e_0,m_) life expectancy, the annual population growth rate (r), male age (p), and the age gap between spouses (g). Three points (*a*–*c*) referenced in Section 1 are labeled on the plot.

Fig. 1 shows what we call the “sustainable” level of polygyny in a given population, i.e., the maximum proportion of men who can be married polygynously, such that all other men of the same age can also marry. For example, in a stable population that is not growing, with a life expectancy at birth (e_0_) of 45 for men and 50 for women, no age gap between husbands and wives, and in which all men seek marriage at age 25, 0.7% of men can sustainably marry 2.5 wives on average (labeled **a** in Fig. 1). This is because the average 25-y-old man can be matched with 1.011 women of the same age (labeled **a** in *SI Appendix*, Fig. S1). Other demographic circumstances yield much higher sustainable levels of polygyny. For example, in a population with 2% annual growth, a male e_0_ of 30, a female e_0_ of 38, and a 10-y age gap at marriage, 52.3% of 45-y-old men can sustainably marry 2.5 wives on average (labeled **b** in Fig. 1). A 2% annual growth rate and a 10-y age gap at marriage can still sustain 12.1% of 45-y-old men marrying 2.5 wives on average, even in a population with a higher male e_0_ of 66 and no female advantage in life expectancy (labeled **c** in Fig. 1).

Overall, the model results demonstrate that, in a wide range of demographic circumstances, polygyny can be practiced without any men in the population being locked out of marriage (Fig. 1). The sustainable level of polygyny varies nonlinearly with the five demographic parameters in the model. Generally, it is higher when 1) women live longer than men, since there will be more prospective brides than grooms; 2) life expectancy is low overall, since women’s usual mortality advantage over men will be concentrated before marriage rather than at older ages; 3) men marry younger women, since the cumulative mortality risk prior to marriage will tend to be lower for prospective brides than for prospective grooms; 4) the population is growing, since men marrying younger women will mean that men are marrying into birth cohorts larger than their own; and/or 5) the likelihood of marrying a second or higher-order wife increases with male age, as the cumulative risk of mortality with age will mean that older men are competing with fewer peers of the same age. Any one of these circumstances contributes to the sustainability of polygyny; in combination, they allow the practice to be sustained at increasing levels. Naturally, the model’s results are contingent on its underlying assumptions (*SI Appendix*, section S2.1), but we emphasize that the model likely underestimates the true level of polygyny that can be sustained in a population, on account of the conservative assumption that marriages are made randomly except with respect to age and sex. Put differently, in the presence of stratification in the marriage market (e.g., along education, wealth, status, and other social dimensions), the level of polygyny that can be sustained is likely to be higher than the value shown in Fig. 1 (*SI Appendix*, section S2.1).

A striking observation that can be drawn from these results is that the demographic conditions identified by the model as increasing the sustainable level of polygyny broadly align with those of many sub-Saharan African populations (*SI Appendix*, section S2.2). Despite considerable variation across countries, sub-Saharan Africa is the region of the world with the highest rate of polygyny, by far (3–5). This observation suggests an interplay between the demographic dynamics under consideration here and the environment, both social and ecological, in line with evolutionary accounts of the stability of different marriage systems across societies (41–44).

## 2. Empirical Analysis

Building on the theoretical results presented in Section 1, we used census data from IPUMS International (45) and IPUMS USA (46, 47) to test whether, in the real world, there is an association between the practice of polygyny and high proportions of unmarried men. We conducted two analyses: one using census data from 30 countries in Africa, Asia, and Oceania in which the marital status of men and women was recorded at the individual level (Section 2.1), the other using data from the 1880 census of the United States, combined with information from the historical record to infer the presence or absence of Mormon polygyny at the county level (Section 2.2).

### 2.1. Global Census Data, 1969–2016.

We used 84.1 million records from 74 censuses that took place in 30 countries between 1969 and 2016 to investigate the within-country relationship between the prevalence of polygyny and the prevalence of unmarried men (*Methods*). This dataset is broadly representative of most of the world in which polygyny was recorded in recent decades (*SI Appendix*, section S3.1).

**Fig. 2.**
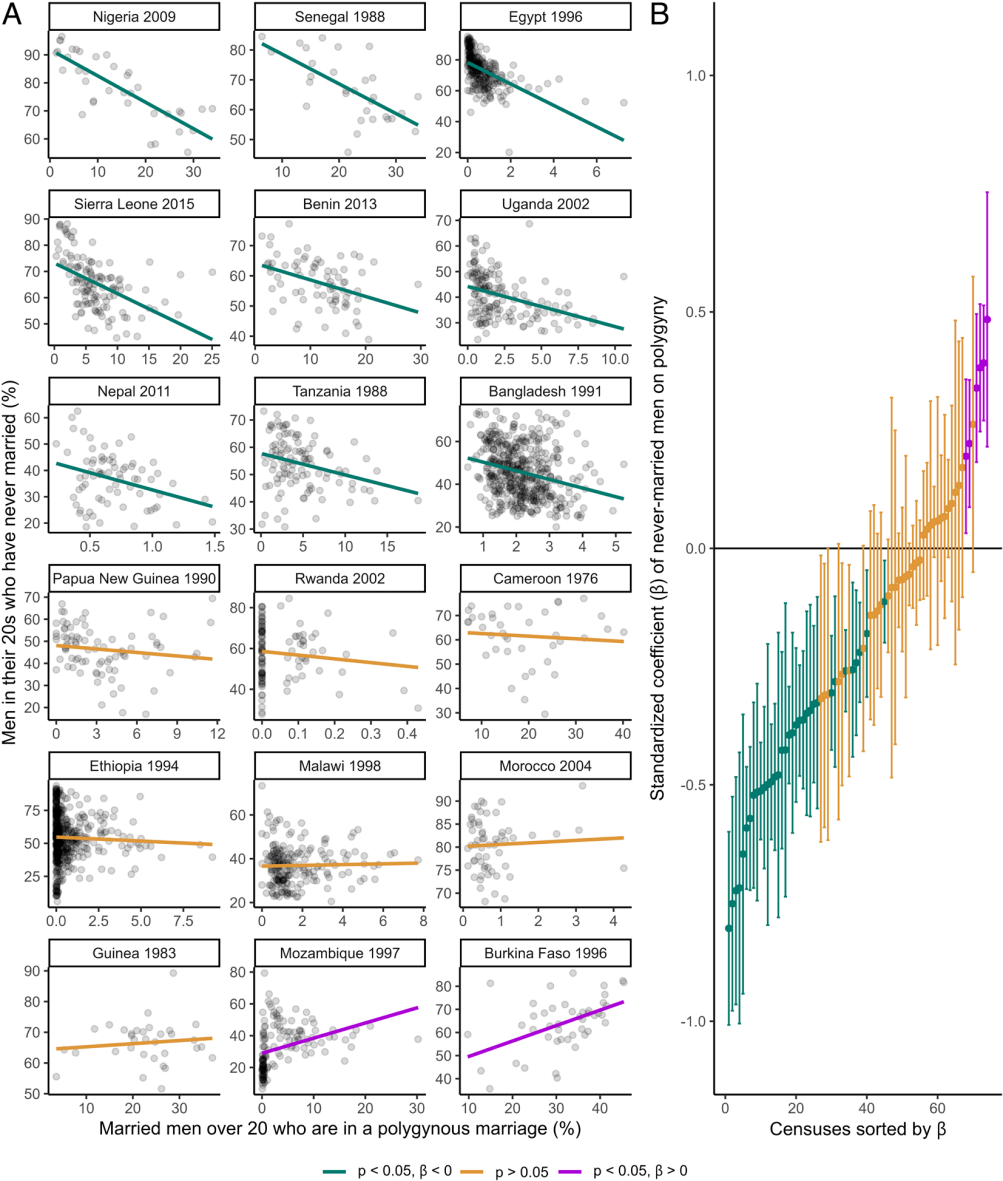
(*A*) Subnational associations between the proportion of married men over age 20 who are in a polygynous marriage and the proportion of men in their 20s who have never been married, in a representative selection of 18 of 74 censuses in the sample, under the main model specification. (*B*) Standardized coefficient (β) and 95% CI of the association in *A* for each of the 74 censuses in the sample, under the main model specification. The statistical significance of the coefficients shown is adjusted for multiple comparisons within the entire multiverse of tests in *SI Appendix*, Fig. S8; the CI shown are unadjusted.

To this end, we conducted a multiverse analysis, corrected for multiple comparisons, yielding a total of 116,424 model specifications. Our main model specification operationalizes the prevalence of polygyny as the proportion of married men over age 20 who are in a polygynous marriage, and the prevalence of unmarried men as the proportion of men in their 20s who have never been married (Fig. 2; see also *SI Appendix*, Figs. S2 and S3). Then, it assesses the association between these two variables with ordinary least squares regression. Under this specification, there is a significant positive association between the prevalence of polygyny and the prevalence of unmarried men at the subnational level in only 6 (8%) of 74 censuses. By comparison, there is a significant negative association in 34 censuses (46%). Therefore, the pattern expected under the assumption that polygyny locks large numbers of men out of marriage obtains in only a minority of cases, whereas the opposite pattern obtains in nearly half. These results do not vary systematically with the mean prevalence of polygyny (*SI Appendix*, Fig. S4), the mean prevalence of unmarried men (*SI Appendix*, Fig. S5), the number of subnational units available (*SI Appendix*, Fig. S6), or the year in which the census was taken (*SI Appendix*, Fig. S7). Nor are they simply driven by differences in the sex ratio within countries: in the 34 censuses which yield a significant negative association, controlling for the local sex ratio in different age ranges attenuates the negative coefficients in Fig. 2 by up to a median of only 18% (*SI Appendix*, section S3.4). Crucially, then, these results are not explained away by variation in sex-specific mortality or migration leading to a feminine skew in the local marriage market.

Focusing on the most recent census for each of the 30 countries, there is a significant positive association between the prevalence of polygyny and the prevalence of unmarried men in only two censuses (7%), namely Mozambique (in 2007, adjusted *P* = 0.042) and South Africa (in 2016, adjusted *P* = 0.004). By comparison, there is a significant negative association in 15 countries (50%). Across the full dataset, there is a significant positive association in four additional censuses: Burkina Faso (in 1996, adjusted *P* = 0.002), Malawi (in 1987, adjusted *P* < 0.001), Mozambique (in 1997, adjusted *P* < 0.001), and South Africa (in 2011, adjusted *P* < 0.001). However, the association is not significant in the other censuses available for three of these countries, namely Burkina Faso (in 2006, adjusted *P* = 0.506), Malawi (in 1998, adjusted *P* = 0.775; in 2008, adjusted *P* = 0.744), and South Africa (in 2001, adjusted *P* = 0.323; in 2007, adjusted *P* = 0.468). Overall, then, we find a significant positive association between the prevalence of polygyny and the prevalence of unmarried men for only four of the 30 countries in the sample, i.e., Burkina Faso, Malawi, Mozambique, and South Africa; except for Mozambique, this positive association does not hold across all censuses available for each country. Therefore, the pattern expected under the assumption that polygyny locks large numbers of men out of marriage is mostly not stable over time, even in the minority of cases in which it does obtain.

The counterintuitive pattern outlined here with reference to the main model specification is robust to alternative operationalizations of key variables, a focus on different age ranges, and implementation of different statistical tests (*SI Appendix*, Fig. S8). Across the 116,424 model specifications applied to the sample, there are more censuses that yield a significant negative association than a significant positive association in 116,396 cases (99.98%). In each of the 28 outlying specifications, most censuses (>68%) show a null association, and across all specifications, the maximum proportion of censuses that show a significant positive association is only 18%. This indicates that, even in those outlying cases, there is no substantial cross-country support for the hypothesis that communities with high rates of polygyny have particularly large numbers of unmarried men.

### 2.2. Census Data for the United States, 1880.

Polygyny was widespread across the Mormon population of the American West at the time of the 1880 US federal census. For example, among all men born in Utah in the 1830s who ever married, nearly 15% engaged in so-called “plural marriage” (48). Using 26.0 million records from this census, combined with historical information about the distribution of Mormon polygyny in the region, we investigated the relationship between this practice and the prevalence of unmarried men across US counties (*Methods*).

**Fig. 3.**
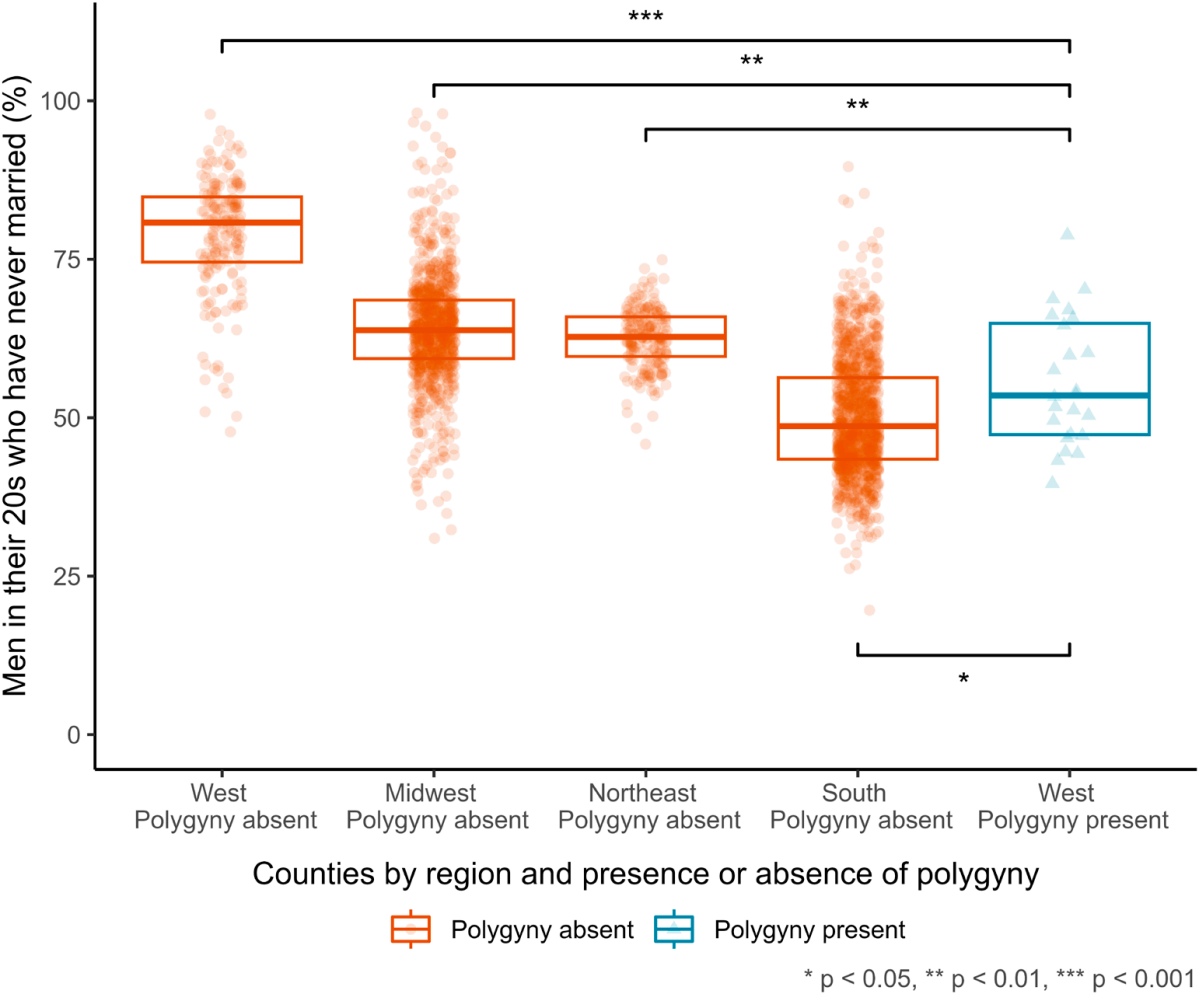
Full distributions, medians, and quartiles of the proportion of men in their 20s who have never been married, across 2,475 US counties as recorded in the 1880 federal census. The counties are disaggregated by the combination of region and the presence or absence of Mormon polygyny.

To this end, we conducted a multiverse analysis, corrected for multiple comparisons, yielding a total of 3,360 model specification. Our main model specification operationalizes the prevalence of unmarried men as the proportion of men in their 20s who have never been married, with 24 of 2,475 counties scored as presenting evidence of Mormon polygyny (21 counties in Utah and three in surrounding states; all located in the West). Under this specification, we used unweighted two-tailed t-tests to perform pairwise comparisons between the 24 counties with Mormon polygyny and other counties, by region (Fig. 3). Results show that the prevalence of unmarried men is significantly lower in those counties than in other counties of the West (n = 191, adjusted *P* < 0.001), and compared to counties of both the Midwest (n = 911, adjusted *P* = 0.002) and the Northeast (n = 215, adjusted *P* = 0.010); conversely, it is significantly higher compared to counties of the South (n = 1,134, adjusted *P* = 0.035). For the most part, then, the observed pattern does not align with the one expected under the assumption that polygyny locks large numbers of men out of marriage: The prevalence of unmarried men is lower, not higher, in counties with Mormon polygyny, compared to other counties in the same region and to counties in two other regions; it is higher only compared to counties in one region. Notably, this pattern is not explained away by the local sex ratio: In 19 of the 24 counties with Mormon polygyny, the prevalence of unmarried men is lower than the average across all other counties with an equivalent ratio of men to women in their 20s (*SI Appendix*, Fig. S9). Finally, this pattern is robust to alternative operationalizations of the prevalence of unmarried men, a focus on different age ranges, and implementation of different statistical tests, as well as to small variations in the scoring of counties for the presence and absence of Mormon polygyny (*SI Appendix*, Fig. S10).

## 3. Discussion

It is commonly assumed that polygyny squeezes large numbers of men out of (heterosexual) marriage markets. A corollary of this assumption is that, as a result, those men may go on to engage in crime and violent conflict, with negative repercussions for both individuals and society as a whole. This view has become influential in political science, evolutionary psychology, and related fields. It has also found its way into the work of development agencies and into the popular press.

However, neither the assumption nor its corollary have been well researched. Therefore, we set out to investigate systematically whether polygyny does indeed lock large proportions of men out of marriage. Through analysis of a demographic model, we showed this will not be the case across a wide range of realistic demographic scenarios. Next, through analysis of over 84 million individual records from 74 censuses for the period 1969–2016 from 30 countries across Africa, Asia, and Oceania, we found that the pattern expected under the assumption that polygyny locks large numbers of men out of marriage is relatively rare, both in absolute terms and in comparison to the opposite pattern: Across countries, a higher prevalence of polygyny tends to be associated with a lower, not higher, prevalence of unmarried men. Finally, through analysis of 26 million individual records from the 1880 federal census of the United States, we found that the average prevalence of unmarried men is lower, not higher, across counties of the West with Mormon polygyny, compared to other counties of the West, and to counties of the Midwest and the Northeast; it is higher only compared to counties of the South.

### 3.1. Explaining the Sustainability of Polygyny.

The determinants of a population’s marriage rate can be roughly divided into supply- and demand-side factors. On the supply side, the sex ratio of the population and the efficiency of matchmaking institutions (including dating apps, in contemporary contexts) determine the mathematical limits of how many marriages can form, and how quickly (49, 50). On the demand side, a large number of cultural, social, and economic forces affect the intensity with which individuals seek marriage, as well as individual preferences about suitable marriage partners (51–53). Supply-side factors do seem to matter in our context: As discussed, despite substantial variation across countries, sub-Saharan Africa presents the highest prevalence of polygyny of any global region, on the one hand, and demographic circumstances that broadly align with those identified by our model as sustaining high levels of polygyny, on the other (Section 1).

Supply-side factors appear to be only part of the explanation, however. Recall that our global census analysis revealed numerous negative associations between the prevalence of polygyny and the prevalence of unmarried men; the fact that these associations largely persist after controlling for the local sex ratio points to an important role for demand-side factors. Specifically, we propose that relatively strong promarriage norms tend to increase the overall rate of marriage in communities with a high prevalence of polygyny to a greater extent than the effect of polygyny on marriage market sex ratios decreases that rate. An analogous dynamic has been noted in the literature linking sex-selective abortion to men’s marriage prospects. Sex-selective abortion of female fetuses, which has been practiced at high rates in China since the 1980s (54), skews marriage markets masculine as the affected birth cohorts age. As in the literature on the effect of polygyny on men’s marriage prospects, it has been assumed that the resulting high numbers of unmarried men would lead to an increase in the crime rate (55, 56). However, there are far fewer unmarried men in China than in neighboring countries, such as Japan and Taiwan (51), which have not historically engaged in sex-selective abortion. This counterintuitive pattern is explained by the fact that China presents much stronger promarriage norms than those other countries. In the Chinese context, the aggregate demand for marriage, rooted in social norms, overrides the relatively small downstream effect of sex-selective abortion on the marriage market.

Accordingly, the available evidence suggests that communities in sub-Saharan Africa with a high prevalence of polygyny have strong pronatalist views—which are, by implication, promarriage. A detailed study of 1980s Kenya finds that for both men and women, the average ideal family size was substantially larger in communities with a high prevalence of polygyny, compared to communities with a low prevalence of the practice (57). A recent study has corroborated this finding across a sample of 29 countries in sub-Saharan Africa, albeit without differentiating between the preferences of men and women (58). Separately, recent evidence of a negative association across 38 countries in the region between the prevalence of polygyny and the proportion of men who are childless at age 40 (59) lends further support to the suggestion of possible link between polygyny and pronatalist norms.

Systematic investigation is required to test the proposed explanation. To this end, we further suggest that differences in rural vs. urban status may also play a role, underpinning the observed variation in both the prevalence of polygyny and the strength of promarriage norms. Available evidence for sub-Saharan Africa indicates that polygyny is more common in rural areas than in urban ones (4) and that urbanization is linked to the weakening of promarriage norms, especially in young adulthood (60), for example, through exposure to education and developmental idealism (61). Should this suggestion hold up empirically, it would imply that urbanization has a stronger effect on men’s marriage prospects than polygyny in itself.

The demand-side explanation proposed here may similarly apply to historical Mormon polygyny. Some researchers have suggested that the practice may have been sustained at high levels by the relatively feminine sex ratios of historical Mormon communities (62, 63). However, this suggestion is at odds with our finding from the analysis of the 1880 US federal census data that, after conditioning on the local sex ratio, the prevalence of unmarried men is lower in most counties with Mormon polygyny, than the average for other counties (Section 2.2). Instead, we suggest that this pattern may have been driven by strong promarriage norms: The nineteenth-century Mormon population is known to have placed relatively high social and moral value on marrying and having children (62, 64). In this regard, our finding of an even lower prevalence of unmarried men across counties of the South, compared to counties of the West with Mormon polygyny, may just be the exception that proves the rule, in the sense that the US South is also known to have been characterized by strong demand for marriage in the late 19th century. Compared to other parts of the United States, in the South there were considerably fewer opportunities for paid work for white women, fostering a reliance on marriage as a means of subsistence (65). Additionally, high male mortality from the 1861–1865 Civil War gave rise to widespread cultural anxiety among women in the South about the prospect of finding a husband; this anxiety was disproportionately greater than the skew in marriage market sex ratios that the war actually caused (66), but it heightened women’s demand for marriage for a sustained period after the war.

### 3.2. Implications for Ongoing Discourse on Polygyny.

To be clear, we are not arguing that polygyny *never* locks men out of the marriage market: Marriage market sex ratios have demonstrable effects on the rates at which men and women marry (50, 67). Rather, we show that it is not warranted, neither theoretically nor empirically, to assume that polygyny necessarily locks large numbers of men out of marriage. Taken together, our results challenge a key foundation of the argument made in much scholarly and popular discourse linking polygyny to social conflict—namely, that polygyny squeezes many men out of the marriage market (15, 20–25). Our theoretical findings show that the intuition driving this foundation is not as solid as is commonly assumed; accordingly, our empirical findings demonstrate that relatively few contexts present the pattern expected under the prevailing assumption.

There are other ways in which polygyny could contribute to conflict. For example, polygyny may fuel class tensions where it is the prerogative of the wealthiest men in a community (6, 68). However, robust causal work is needed to test such a claim, especially due to the risk of confounding: The degree of wealth inequality in a population can influence both the prevalence of polygyny (44) and the likelihood of conflict (69). As noted, existing empirical work linking polygyny to conflict presents serious shortcomings (*Introduction*), and we contend that it has not taken the risk of confounding seriously. For example, it seems plausible that rural areas furthest removed from the influence of norms favoring monogamy are also furthest away from institutions and other forces (e.g., economic) which contribute to the reduction of armed conflict. As discussed, recent work has shown that posited associations between polygyny, child health, children’s education, and intimate partner violence may be explained away, in large part, by confounding factors (11–13); the same may be true of the posited association between polygyny and conflict.

Finally, we highlight the implications of our work for two strands of discourse informed by evolutionary thinking. First, our work challenges the argument that monogamy emerged, over evolutionary time, through a process of (cultural) group selection (e.g., 15, 27). To the extent that monogamy reduces societal instability—by lowering the proportion of unmarried men, compared to polygyny, thus mitigating the potential for within-group conflict among men—it has been theorized that its adoption enabled success in between-group competition. By casting doubt on the widespread assumption that a high prevalence of polygyny inevitably involves large proportions of men going unmarried, our findings undermine a key premise of this argument.

Second, our work challenges the view that the interplay of “effective polygyny” and men’s evolved psychology gives rise to the “incel” movement’s desire to restrict women’s sexual autonomy. The term “incel” is a *portmanteau* of the words “involuntary celibate”, and the incel movement is an online misogynist subculture that has occasionally been linked to acts of terrorism (70). The term “effective polygyny” reflects the belief in this movement that a small number of men (referred to as “Chads”) acquire a large number of sexual partners, leaving few partners for other men (71–73). This belief closely parallels the assumption, discussed throughout this article, that polygynous marriage in e.g., sub-Saharan Africa locks many men out of the marriage market, and in the context of the incel movement, this belief has already been lent credibility by the work of some evolutionary psychologists (71). Empirical work already suggests that the rise of the incel movement has not, in fact, coincided with a rise in “Chads”. For example, sexual survey data from the United States between 2000 and 2018 shows that the increase in the proportion of men who report having no sex in the previous year is not mirrored by a corresponding increase in the proportion of men who report having three or more sexual partners over the same period (74). Moreover, our results urge caution in drawing simplistic conclusions about what effect polygyny really does have on men’s marriage or mating prospects.

## 4. Conclusion

In closing, we emphasize that our work should not be taken as a defense of, nor a justification for, polygyny nor any other marriage system. Instead, our results expose the flawed logic that polygyny inevitably creates large proportions of men with no hope of ever marrying, highlighting the complexity of marriage as a social institution. On this point, we note that criticisms of polygyny come disproportionately from the Global North, and they typically ignore counterarguments by anthropologists, lawyers, philosophers, and others, pointing out that, for example, many of the harms to women attributed to polygyny also apply under monogamy (75–77). More broadly, structural oppression and interpersonal violence against women can exist under any marriage system, including polyandry (78). Humans have long exhibited great variation in marriage systems, and analogous family forms often have disparate meanings, and effects, in different social contexts (1, 2). This complexity implies that policies aimed at regulating marriage and the family, or otherwise impacting these key aspects of human social life, must be correspondingly nuanced—including policies aimed at promoting the rights of women. In this regard, an insightful complement to our work comes from the literature linking family forms to child poverty. It has long been assumed that childbirth out of wedlock is a driver of adverse outcomes for children in the United States, but recent work shows that the relationship between the two is simply confounded by socioeconomic disadvantage and other structural factors (79, 80). In this example, simplistic assumptions about marriage and family systems have impaired scholarly progress, advancement in public discourse, and the extent to which policy can promote human flourishing. Our work strongly suggests that this may also be true in the case of polygyny.

## 5. Methods

### 5.1. Demographic Model.

We used a life-table approach to model the theoretical effect of polygyny on the proportion of unmarried men in a population. Specifically, we calculated the ratio of women to men at different ages—so-called “availability ratios” (e.g. 38)—according to a range of demographic factors. We assumed a population with five fixed parameters: no migration, a stable population regime, a sex ratio at birth of 1.05, sex- and age-specific mortality rates in line with the UN’s general model life tables (81), and polygynous men marrying 2.5 wives on average. Next, we compared the number of women to men at specific male and female ages at which marriages are likely to exist, as a function of the following five variables: male life expectancy, the difference in life expectancy between men and women, the population growth rate, male age, and the age gap between spouses. The sex ratios at marriageable ages under these different variables were calculated by scaling the *l_x_* column in the appropriate male and female life tables by the sex ratio at birth and population growth rate as needed. We then calculated the proportion of men who needed to marry 2.5 wives on average such that the remaining number of men equaled the remaining number of women; this step converted the sex ratios at a given marriageable age, shown in *SI Appendix*, Fig. S1, to the “sustainable” level of polygyny, shown in Fig. 1, defined as the proportion of men of that age who can be married polygynously, such that all other men of the same age can still marry monogamously. A detailed discussion of the model’s assumptions, including their applicability to the sub-Saharan African context, is in *SI Appendix*, section S2.

### 5.2. Analysis of Global Census Data.

We investigated the relationship between the prevalence of polygyny and the prevalence of unmarried men, at the subnational level, using data from IPUMS International, a global repository of demographic microdata based at the University of Minnesota (45). We analyzed 84.1 million person-records grouped into 11,943 localities from 74 censuses conducted between 1969 and 2016 across 30 countries. By region, the censuses in the sample are distributed as follows: 52 from 20 countries in sub-Saharan Africa, 11 from five countries in North Africa and the Middle East, eight from four countries in South and Southeast Asia, and three from one country, Papua New Guinea, in Oceania.

Using these data, we assessed the bivariate association between the prevalence of polygynous marriage and the prevalence of unmarried men. In our main model specification, we used ordinary least squares regression to test the association between the proportion of all married men over age 20 in a polygynous marriage and the proportion of men in their 20s who have never been married. This was our main specification because the literature linking polygyny to conflict seems to imply a particularly strong risk of conflict if young men are squeezed out of the marriage market; this may be due to the association between youth and violent crime (82). However, we also tested for associations between polygyny and unmarried men using alternative statistical tests, alternative operationalizations of our explanatory and outcome variables, and alternative age ranges in which to measure those variables, yielding a total of 116,424 model specifications. We did so out of recognition that there is a multiverse of ways in which those variables can be operationalized and correlated using the same underlying data (83). Given the large number of regressions conducted, we used the Benjamini–Hochberg method (84) to control for multiple comparisons in all analyses. Further details of the sample selection criteria and other analytical decisions are in *SI Appendix*, section S3, with a discussion of the assumptions and limitations of the analysis.

### 5.3. Analysis of US Census Data.

IPUMS USA’s full-count (100%) sample of the 1880 census of the United States (46, 47) covers all of the contiguous United States except for the Native American reservations of the time and the land of present-day Oklahoma (85, 86). The 1880 census is uniquely well-suited to studying the relationship between polygyny and the prevalence of unmarried men, and we are able to overcome its main limitation—that polygyny cannot be reliably identified at the household level—by drawing on the historical record to score counties for the presence or absence of Mormon polygyny (*SI Appendix*, section S4).

After restricting the records of the 1880 census to only include individuals aged 20 or older, and counties with 100 or more adult men recorded, we were left with a dataset of 26.0 million individuals across 2,475 counties. We tested for a difference in the proportion of unmarried men by marriage system under a range of model specifications. In our main specification, we used unweighted t-tests to compare the proportions of men in their 20s who had never been married—between counties we had labeled as polygynous vs. monogamous, with the latter disaggregated by the four main regions of the United States (as currently defined by the US Census Bureau). As in our global analysis, we also tested a large number of alternative model specifications (3,360) and have reported all tests of significance after being corrected for multiple comparisons. Further details of sample selection criteria and other analytical decisions are in *SI Appendix*, section S4, with a discussion of the assumptions and limitations of the analysis.

## Supplementary Material

Appendix 01 (PDF)

## Data Availability

The census microdata obtained from IPUMS International and IPUMS USA cannot be redistributed, but it can be downloaded at no cost after registering at https://www.ipums.org/ (45–47). To improve reproducibility, we have made the following materials available in an OSF repository (https://osf.io/tgb3k) (87): the UN model life tables used to produce the demographic model, the codebooks of the IPUMS extracts we use, and the R Markdown files used to analyze those extracts and produce the figures in the main text and supplement.
