# Supplementary material for: High rates of polygyny do not lock large proportions of men out of the marriage market
Source: Proc Natl Acad Sci U S A. 2025 Oct 3;122(40):e2508091122. doi: 10.1073/pnas.2508091122 (PMC12519187; doi:10.1073/pnas.2508091122)
Supplement: Supplementary file 1 — Appendix 01 (PDF) [file pnas.2508091122.sapp.pdf]

High rates of polygyny do not lock large proportions of men out of the marriage market

Hampton Gaddy, Rebecca Sear, Laura Fortunato

Supplementary discussion **1**
S1. Limitations of past research on polygyny and conflict 1
S2. Further details on the formal demographic model 2
S2.1. Model assumptions 2
S2.2. Comparison of model assumptions and results to Sub-Saharan Africa 4
S3. Further details on the global census analysis 5
S3.1. Sample selection 5
S3.2. Main statistical analysis 6
S3.3. Limitations 7
S3.4. Controlling for local sex ratios 8
S4. Further details on the US census analysis 8
S4.1. Sample selection 8
S4.2. Statistical analysis 9
Supplementary figures **11**
Figure S1 11
Figure S2 12
Figure S3 13
Figure S4 14
Figure S5 15
Figure S6 16
Figure S7 17
Figure S8 18
Figure S9 19
Figure S10 20
Supplementary tables **21**
Table S1 21
Table S2 24
Supplementary references **27**

Supplementary discussion

S1. Limitations of past research on polygyny and conflict

To our knowledge, the only systematic test of the association between polygyny and the proportion of the men in a population who are unmarried appears in Table 6 in the supplementary materials of Henrich, Boyd, and Richerson's "The puzzle of monogamous marriage" (1). As the authors describe it: "To show that increasing polygyny is associated with a higher percentage of unmarried men, we use national-level data from Kanazawa and Still, who compiled crime statistics, demographic information and economic data together with a measure [sic] degree of polygyny for 157 countries. To create a measure of polygyny, Kanazawa and Still coded all of the cultures in the Encyclopedia of World Cultures on a four point scale (from 0 = monogamy is the rule and is widespread, to 3 = polygyny is the rule and is widespread), and then developed a country-level value by aggregating all of the cultures within a country, multiplying the values for each culture by the fraction of the country's population represented by that culture" (p. S11).

This analysis has several empirical and theoretical weaknesses in its ability to draw a convincingly causal link between polygyny and unmarried men. The authors' preferred regression model supports the often-assumed positive association between polygyny and unmarried men at the $p < 0.10$ level (estimated $p = 0.0945$) but without a correction for multiple comparisons. Correcting for multiple comparisons by applying the Benjamini–Hochberg method (2) to the 28 tests of coefficient significance conducted in Table 6 of the authors' supplementary materials increases the p-value for the positive relationship between polygyny and unmarried men of this preferred model to an estimated 0.1161 (results not shown). In addition to the weak evidence for statistical significance in this analysis, there are a number of significant analytical limitations in the Kanazawa and Still dataset and its analysis by Henrich, Boyd, and Richerson: the likely omitted variable bias of the cross-country design, the ordinal measurement of polygyny, the temporal disconnect between the data collected on polygyny and proportions of unmarried men, and the generalization from the ordinal level of polygyny suggested by the anthropological record for a country to the level of the whole country.

Turning to the claim in past empirical work that polygyny causes negative social outcomes because it creates a pool of men excluded from the marriage market: there are a number of empirical challenges to the literature that has tried to test this hypothesis. Cross-sectional analyses do not necessarily face the risk of reverse causality that many researchers dismiss them as facing (3), but in this case, there is a credible argument that adverse social outcomes like war or despotism might cause high rates of polygyny under certain circumstances, rather than vice versa. Two scholars have argued for this opposite causal interpretation in the past (4, 5). Meanwhile, other work suggests that the observed association between polygyny and war is due to a confounding effect of patriarchy (6). We add the possibility that resource concentration amongst societal elites could be another confounding factor, as social inequality both contributes to women's decision-making in a polygynous marriage market (under many but not all ecological regimes; see (7)), and social inequality can foster social unrest. Some behavioral ecologists also dispute the idea that large numbers of unmarried young men will necessarily commit violence in large amounts (8), and the analytical validity of one key study linking polygyny to conflict has been called into question on several empirical and theoretical fronts (9). Additionally, a recent study that is the first to attempt to robustly identify a relationship between marriage market squeezes

against men and men joining armed groups (10) faces empirical problems in its use of rainfall as an instrumental variable for marriage market conditions (see 11), and in its use of aggregated news reports to measure the prevalence of conflict (see 12). The exclusion restriction of the instrumental variable analysis requires that rainfall that does not affect conflict through any channel besides its effect on marriage markets, but this is unlikely since rainfall is associated with conflict in non-polygynous contexts (13) and rainfall is often used as an instrument for the effect of income and other factors on conflict (14).

S2. Further details on the formal demographic model

S2.1. Model assumptions

As discussed in Section 1 and in the *Methods*, we modeled the relationship between the rate of polygyny and the proportion of unmarried men in a demographically stable population that is closed to migration. We set the sex ratio at birth to 1.05; adopted sex- and age-specific mortality rates from the UN's general model life tables (15); and set the average number of wives for polygynous men to 2.5, based on observations from Sub-Saharan African populations (16–20).

Against these fixed parameters, we calculated what proportion of men in the population could marry polygynously such that all other men of the same age could marry at least monogamously, as a function of five key demographic variables: the life expectancy at birth of men and women, men's age, the age gap between spouses, and the population growth rate. We term this proportion the "sustainable" level of polygyny. Our modeling strategy dispenses us from specifying rates of marriage, divorce, widow(er)hood, and remarriage, thus ensuring tractability of the model (see *Methods*). The total ratio of women to men at specific ages can be taken as the average number of women available for each man to be married to within those age pairings, as long as the following assumptions hold: roughly equal proportions of men and women want to marry heterosexually; marital unions are formed randomly, except with respect to age and sex; the likelihood of remarriage does not differ considerably by sex; and remarriage happens on average a negligible time after divorce or widow(er)hood.

Departure from any of the model's assumptions may lead to a deviation from the sustainable levels of polygyny presented in Figure 1, in either direction. In this section, we will now discuss the effects of specific deviations from the model assumptions. For example, a decrease in mortality and/or fertility reduces the ratio between the sizes of younger female cohorts and older male ones, making marriage market sex ratios less feminine, such that the model overestimates the sustainable level of polygyny. Similarly, an increase in the sex ratio at birth—which may occur with mortality decline (21, 22)—will increase the ratio of men to women at all ages, resulting in less feminine marriage market sex ratios. Meanwhile, the assumption of no migration means that the model systematically underestimates the sustainable level of polygyny in many rural areas; for economic and cultural reasons, rural-to-urban migration, e.g., in Sub-Saharan Africa is often skewed male, leaving rural populations with a relatively strong feminine skew in their sex ratio (23–26). The average number of wives for polygynous men may be lower or higher than 2.5—leading to a sustainable level of polygyny that is, respectively, higher or lower than the one calculated under the current assumption.

The assumption that marriages are made randomly, except with respect to age and sex, means that the model underestimates the sustainable rate of polygyny in some

marriage markets. Individual preferences about suitable marriage partners can be thought of as splitting the marriage market for a whole community into several smaller ones, which we assume to be mutually exclusive, for the sake of simplicity. The sex ratio of some of these “sub-markets” will differ from the sex ratio of the overall market. For example, many less-educated men in contemporary India self-select into a highly masculine sub-market, by virtue of their preference for marrying women with a lower level of education than themselves, despite the rapid advancement in women's education in the country. The exclusion of educated women from this sub-market leads to a strong feminine skew in the sub-market for more highly educated men (27).

Polygyny is uncommon in India (see 28), but in this context, it is clear that polygyny could be practiced sustainably in the highly feminine sub-market in which more-educated men compete, without affecting the chances of marrying for the less-educated men in the highly masculine sub-market. Mathematically, the sustainable level of polygyny will be higher than the values presented in Figure 1 whenever a marriage market with a feminine sex ratio can be split into several semi-independent sub-markets, some of which have a masculine sex ratio. For example, consider a population of 1,100 prospective brides and 1,000 prospective grooms (a sex ratio of 1.1), which can be split into two distinct sub-markets, one with a sex ratio of 1.4 (700 women to 500 men), the other with a sex ratio of 0.8 (400 women to 500 men). If the sub-markets were combined, only 33 men (6.7% of men in the whole population) could sustainably marry 2.5 wives on average. With the sub-markets separated, 133 men (26.7% of men in the feminine sub-market, corresponding to 13.3% of men in the whole population) can sustainably marry 2.5 wives on average. The “additional” 100 men who are able to marry polygynously in the feminine sub-market corresponds to the 100 men in the masculine sub-market who are not able to marry, on account of there being only 400 women to 500 men in that sub-market. Crucially, these men are not able to marry on account of their own preferences for suitable partners: they are not prevented from doing so by men in the other sub-market who marry polygynously. Yet by adopting their restrictive marriage preference, they enable other men in the population to marry polygynously.

Extending the model to capture assortative and disassortative marriage with respect to e.g., education, ethnicity, class, wealth, etc., can provide further insight into this dynamic, but doing so is beyond the scope of the present study. A further extension, which is also beyond the scope of the present study, is to investigate how individual marriage preferences change over time—and therefore how individuals move between different marriage markets—and how membership of multiple sub-markets affects an individual’s marriage prospects. This may be particularly interesting to consider in future work because there is evidence that individual marriage preferences are highly malleable, such that they often become less restrictive in the face of a marriage market squeeze (29–32); this malleability may increase men’s ability to find a spouse in the presence of a marriage squeeze caused by other men’s polygyny. However, we emphasize that the mathematical argument for why our model tends to be conservative holds for any particular cross-section of time: while preferences about suitable marriage partners may change over time, they are fixed for a given population at a specific moment. These preferences sort individuals in the population into distinct marriage sub-markets, giving rise to the dynamic outlined above.

A final set of model assumptions relates to remarriage following divorce or widow(er)hood. To reiterate, the model assumes that the likelihood of remarriage is comparable between the sexes, and that any delay between marital dissolution and remarriage is negligible. A possible deviation from this assumption could involve remarriage being less socially acceptable for women than for men, such that women do not always

remarry or do not remarry shortly after divorce or widowerhood. In that case, women are effectively "removed" from the marriage market—permanently, if they are not allowed to remarry, or temporarily, if they do eventually try to marry again. As a result, the sex ratio of the marriage market would skew less feminine than shown in Figure S1, reducing the sustainable levels of polygyny below the values in Figure 1.

At the same time, the way in which we model the sustainability of polygyny is systematically conservative in populations in which men are content with having been married at least once. Divorce has no effect on the results presented in Section 1 because the sustainability of polygyny is calculated in terms of the proportion of men at certain ages who can be married at a particular point in time, mathematically speaking, not in terms of the proportion of men who will ever be or ever have been married. Therefore, a woman getting divorced and then remarrying another man has no effect on the proportion of men currently married; what may be affected is the proportion of men ever married, if she takes a never-married man as her spouse after the divorce. Our work is primarily meant to engage with the research question of whether polygyny increases the proportion of unmarried men in a population such that polygyny causes societal instability. In our modelling approach, we engage with this question by estimating the "sustainable" level of polygyny as the maximum level of polygyny by a group of men such that all men of the same age can be married at least monogamously at the same point in time. However, if men are fairly content with simply having been married at some point, i.e., if being divorced or widowed does not cause them to pose same "risk" to social stability as being locked out of marriage in the first place, then the true "sustainable" level of polygyny may be considerably higher than the values we estimate in Figure 1. This is particularly worth considering given that polygynous marriages tend to result in divorce more often than monogamous ones in the same community (33–35); one can think of this as creating a high circulation among men, over time, of women formerly in polygynous unions.

S2.2. Comparison of model assumptions and results to Sub-Saharan Africa

Notably, the model's assumptions and the variables shown by the model to increase the sustainable level of polygyny provide a particularly good fit to the demographic circumstances documented in Sub-Saharan Africa, the region of the world in which polygyny is most common (36). As noted, the assumption that polygynous men marry 2.5 wives on average is based on observations from Sub-Saharan African populations (16–20). Furthermore, Sub-Saharan African populations have a lower life expectancy than much of the rest of the world (37), even after ignoring the effect of HIV/AIDS (38, 39)—coupled with the lowest sex ratios at birth in the world (21, 40) and with higher longevity for women than men, as is the case in almost all populations (41, 42). Sub-Saharan Africa is also the global region that has and will have the highest natural population growth by far this century (43). Meanwhile, polygynous communities in Africa are strongly characterized by a large age gap at marriage between men and women (16, 44, 45), with men entering higher-order marriages as they get older (16–18, 46, 47). Additionally, women in many Sub-Saharan African populations remarry at high rates following divorce or widowerhood (48–51), and contemporary rural-to-urban migration rates in the region are much higher for men than women (52, 53). Anecdotally, some communities in West Africa justify the institution of polygyny on the grounds that there are many times more women in the world than men (54); while the intuition underlying this folk explanation is demographically incorrect, our model

results suggest that the demographic circumstances of populations in this region may play a key role in sustaining polygyny at cross-culturally high levels.

S3. Further details on the global census analysis

S3.1. Sample selection

IPUMS International contains individual-level microdata from a total of 80 censuses in which polygynous marriages are directly reported in the data (55). Individuals can then be grouped at the lowest-level administrative area possible, in order to analyze the sub-national association between the prevalence of polygyny and the prevalence of unmarried men across administrative areas.

IPUMS International also contains individual-level microdata from 5 waves of the General Household Survey of Nigeria. We have included these survey waves in our global analysis, bringing our global sample to 85 sets of microdata. We include the Nigerian data in our analysis because it is in the same standardized format as the IPUMS census data; it also reports polygynous marriages directly in the data; and it provides a representative window into the high-polygyny, high-population context of Nigeria. However, there are two aspects of the Nigerian data that are distinctive. First, the purpose and size of this survey is different from that of a traditional census, i.e., an enumeration of the full population. The 2006–10 waves used in our analysis were designed for collecting data on economic and health behavior variables, and to that end, they only sampled 0.5–0.7% of the total national population, depending on the wave. However, the survey was conducted using random sampling that should allow unbiased inference of the proportion of men who are unmarried or practicing polygyny at the local level; the survey sampled 10–15 random households in a number of districts (specifically, enumeration districts from the 1991 census) that were randomly selected from within each Nigerian state. Second, we grouped the individual-level records at the state level for our subnational analysis, even though the lowest-level administrative area available in the 2007–10 waves is the local government area (LGA). This is because the small overall size of the survey (0.5–0.7% of the total national population) and the large number of LGAs in existence mean that there are few people represented in most LGAs. Therefore, estimates of the prevalence of polygyny and unmarried men are much more reliable at the state level than the LGA level.

Contemporary demographic microdata usually does not comprise all the individual-level records from the given census, primarily for privacy reasons. For each of the 80 censuses and 5 Nigerian survey waves (hereafter, 85 “censuses”) that record polygyny, IPUMS International holds a random sample of the households recorded, ranging from a 0.05% sample to a 16.6% sample of households, depending on the census. Tables S1 and S2 provide some relevant summary statistics about these censuses. We applied reasonable exclusion criteria to those 85 censuses. First, we only included in our analysis individuals aged 20 or older for whom sex and marital status were recorded. Then, for each census, we grouped individuals’ records by sub-national administrative area (hereafter, “locality”), and then we excluded all localities with fewer than 100 men aged 20 or older recorded in the microdata. Then, we excluded all censuses with fewer than 25 localities represented—such as the 2008 census of South Sudan, in which respondents were only coded as living in one of 10 states. This series of exclusion criteria restricted the number of censuses in our global sample from 85 to 74.

Census microdata is not available for some countries with a high prevalence of polygyny, such as Chad, Niger, and the Democratic Republic of the Congo (36, 56). These countries are therefore not included in our dataset. We also recognize that the conceptualization of polygyny and the consequent extent to which it is captured in standard demographic sources varies over time and place (57), and we note that longitudinal data gives a better sense of the life-course prevalence of polygyny than cross-sectional data (58). However, we believe that our data allows adequate reckoning of how relatively common polygyny is at the sub-national level (for the purposes of our analysis), and that our data is representative of the majority of populations in which polygyny has been practiced in the recent past.

S3.2. Main statistical analysis

The results of our main model specification are summarized in Figure 2. In this specification, we estimate the sub-national association between the proportion of married men over age 20 who are in a polygynous marriage and the proportion of men in their 20s who have never been married using ordinary least squares regression. However, a skeptical reader might inquire whether the results in Figure 2 are highly sensitive to the operationalisation of the prevalence of polygyny, the operationalisation of the prevalence of unmarried men, the age bands in which we measure those two prevalences, or the statistical test used to assess the association. To that end, we conducted a multiverse analysis corrected for multiple comparisons.

In each specification, we operationalized the prevalence of unmarried men as alternatively (i) the proportion of men who are single or (ii) the proportion of men who are single or divorced. Then, we operationalized the prevalence of polygyny as alternatively (i) the proportion of married men who are polygynously married, (ii) the proportion of all men who are polygynously married, (iii) the proportion of married women married to a polygynyous man, (iv) the proportion of all women married to a polygynyous man, (v) the proportion of married women who are a second or higher-order wife, or (vi) the proportion of all women who are a second or higher-order wife. Then, we measured the prevalence of unmarried men in one of 21 age bands: eight 5-year bands (20–24, 25–30, … 55–59), seven 10-year bands offset by 5 years each (20–29, 25–34, … 50–59), five 20-year bands offset by 5 years each (20–39, 25–44, … 40–59), and at all ages 20 or older.[1] Then, we measured the prevalence of polygyny in one of those 21 age bands, chosen independently from the unmarried-men age band. Then, we assessed the significance of the resulting association between polygyny and unmarried men using (i) ordinary least squares regression, (ii) logistic regression, (iii–v) least squares regression weighted by the total size of the locality's population aged 20 or older, by the size of the male population in the age range within which

[1] We did not conduct analyses using narrow age bands above the age of 59, because there are decreasing numbers of people represented in our data at older ages. For example, the age structure of Nigeria means only 5% of the population was over the age of 59 in 2010. Then, of that 5% of the population, we only observe the proportion contained in IPUMS International's random sample of the underlying census, and then, for the sake of our subnational research design, we would have to split the observed adults over the age of 59 into at least 20 subnational localities. Therefore, we believe that the resulting estimates of the prevalence of polygyny or unmarried men at older narrow age ranges would be unreliable. We also believe that the decision not to closely examine the polygyny of men age 60+ (except as part of the overall age 20+ age band) does not have a substantial bearing on our research question, since the number of proportion of adult men age 60+ in, for example, most Sub-Saharan African population is so small that, even if they are intensely polygynous, their polygyny will not have a large effect on the sex ratio of the overall marriage market.

the prevalence of unmarried men was assessed, or by the size of the (male or female) population in which the prevalence of polygyny was assessed, (vi–viii) logistic regression weighted by the same three sets of weights, and (ix–xi) three unweighted non-parametric tests (rank regression, Theil–Sen regression, and Siegel regression). Some IPUMS samples provide individual-level weights that are designed to improve the representativeness of aggregate calculations; where these weights were available, we additionally tested the effect of applying or not applying them when estimating the prevalence of polygyny and unmarried men at the locality level.

Allowing for all combinations of these different analytical decisions results in 116,424 model specifications (including our main specification), and we applied each model specification to each of the 74 censuses in our global dataset. In 18.6% of the specifications, at least one census was dropped due to no polygyny being observed in the given age range in which it was being measured. In the most extreme case, 13 censuses were dropped for this reason, yielding a minimum number of censuses to which any specification was applied of 61. Overall, a total of 8.58 million regressions were conducted (116,424 specifications applied to an average sample size of 73.7 censuses). Then, we used the Benjamini–Hochberg method to control for multiple comparisons (2). The relationship between different measures of polygyny and different measures of unmarried men in the same locality means that within-census tests are not independent, but the fact that those measures overwhelmingly correlate with each other positively (see replication file) means that the Benjamini–Hochberg method is warranted, as opposed to the more conservative Benjamini–Yekutieli method that allows for negative dependency (59). As a result of this method, the estimated false discovery rate is below the level of statistical significance chosen, and so we use the standard $p < 0.05$ level for assessing significance.

S3.3. Limitations

The global analysis assumes that the geographic boundaries of marriage markets match those of the localities in our analysis—rather than crossing locality borders or being stratified within localities by ethnicity, for example. However, accounting for the spatial autocorrelation that marriage market spillovers create would only increase our standard errors (see e.g., 60), meaning that even more censuses would return a null association between the prevalence of polygyny and the prevalence of unmarried men. Also, we find numerous significant negative associations irrespective of the number of sub-national units of analysis available to us (Figure S6), i.e., irrespective of roughly how large or small we assume local marriage markets to be.

Another area for caution comes from the risk of mis-counting polygynous men–and particularly the risk of under-counting them. In order to utilize the widest set of IPUMS samples, we use the POLYMAL variable to identify men who are polygynous and the POLY2ND variable to identify women who are the second- or higher-order wife of a polygynous man. The construction of these variables depends on the IPUMS family interrelationship algorithm, which has deficiencies in identifying polygynous men who are not the head of their household or who do not co-reside with two or more of their wives (61). However, the levels of polygyny that we observe (Table S2) are in a broadly credible range, and we are not aware of any theoretical reason why this linkage failure would bias the key patterns we report. In order for it to undermine the credibility of the significant negative associations in Figure 2, the proportion of married men who are polygynous would have to be disproportionately underreported in communities in which a relatively high proportion of

men are married. It seems plausible for the number of polygynous men as a proportion of *all* men to be underestimated in communities with comparatively high male marriage rates—because there are more opportunities for men's linkage to their wives to fail—but our multiverse analysis shows that our headline finding is robust to the relevant measure of the prevalence of polygyny used (i.e., the proportion of all men vs. the proportion of married men only). For linkage failure to bias the association in Figure 2, polygynous men would have to disproportionately reside with only one of their wives, and/or reside with other male heads of household, in places in which male marriage rates are systematically high, within the countries in our sample.

S3.4. Controlling for local sex ratios

One possible explanation for the negative association between polygyny and unmarried men in our global sample is that relatively polygynous communities have much more feminine sex ratios than relatively monogamous communities. This could occur for some combination of three reasons: 1) communities have feminine sex ratios for some exogenous reason and then polygyny rates rise in response to the sex ratio; 2) communities could practice polygyny and then unmarried men emigrate from the community to seek marriage where the sex ratio is more feminine; or 3) there is a correlation between polygyny and feminine sex ratios by chance or due to confounding by an independent third factor. If one of these phenomena or some combination occurs, polygyny's effect of skewing marriage market sex ratios masculine could potentially be dwarfed by the overall feminine sex ratio of the population, and consequently, marriage markets in polygynous communities would be more favourable to the average man than marriage markets in monogamous communities.

To test whether variation in sex ratios explains the negative associations reported in Figures 2, we added the local sex ratio at ages 20–29 to the ordinary least squares regression underlying our main model specification. Doing so only has modest effects. In Figure 2, we report 34 significant negative associations, 34 null associations, and 6 significant positive associations. When controlling for the local sex ratio at ages 20–29, we find 26 negative associations, 41 null associations, and 7 significant positive associations; nine associations that were originally significant and negative became null, one association that was originally null became significant and negative, and one association that was originally null became significant and positive. When focusing on the 34 associations that were originally significant and negative, controlling for the sex ratio at ages 20–29 only weakens the point estimates of the coefficient of the association between polygyny and unmarried men by a mean of 27% and a median of 18%. Moreover, controlling for the sex ratio at other ages does not result in a larger mean or median reduction in the strength of the negative associations. We tested the effect of controlling for the male-to-female sex ratio in all 21 of the age bands listed in Section S3.2 (i.e., 20–24, 25–30, … 55–59; 20–29, 25–34, … 50–59; 20–39, 25–44, … 40–59; and at all ages 20 or older), and controlling for the sex ratio at ages 20–29 had the largest effect. These results are shown in our replication file.

S4. Further details on the US census analysis

S4.1. Sample selection

We used the 1880 federal census of the United States to examine the case of Mormon polygyny for several reasons. In general, studying Mormon polygyny is valuable because it has been the subject of studies reporting that polygyny increases social and

biological competition among men (1, 62–65). Specifically, 1880 saw the last national census before the systematic prosecution of Mormon polygyny with the Edmunds Act of 1882 and the abandonment of polygyny by the mainstream Mormon church in 1890. The 1880 census is also the only set of national microdata that is appropriate for studying this question, in any case. The 1880 census was the first US federal census to ask respondents their marital status. Then, almost all the returns of the 1890 census were destroyed in a fire or due to subsequent bureaucratic negligence (66). The full-count 1900 census is available with marital status recorded, but the population of Utah nearly doubled between 1880 and 1900 in a way that renders the 1900 census unhelpful; the population growth was mostly due to migration from out-of-state, i.e., from people who never lived in a polygynous community.

S4.2. Statistical analysis

The 1880 census was the first federal census in the United States to report an individual's relationship to the designated head of their household (67). Therefore, the prevalence of polygyny in relevant communities can be estimated as the proportion of adult men recorded as having more than one wife living in their household. However, we did not opt for this approach. Polygynous men's wives often resided in different households (68), but the men themselves could only be recorded in one household at the census. In one part of Utah, at least 39% of polygynous men had at least one wife who was not reported as living in the same household as them in the 1880 census (69, 70). Indeed, the key attempts to estimate the prevalence of polygyny in nineteenth-century Mormon communities have all involved linking census records to Mormon church records and genealogical sources (68–73). Lacking access to this data, we scored each county for the presence or absence of Mormon polygyny based on historical information about the distribution of the Mormon population in the West, given that polygamy (called "plural marriage") was a fundamental tenant of the mainstream Mormon church at the time (69, 74). In the late nineteenth century, the Mormon population was almost entirely confined to the state of Utah—where, in 1880, an estimated 82% of the state population was Mormon, and at least 57% of each county's population was Mormon (75)—and the Mormon population was otherwise heavily concentrated in one county each in the states of Idaho (Oneida County), Arizona (Apache County), and Nevada (Lincoln County) (71, 76–78).

After scoring counties accordingly as polygyny present vs. absent, we conducted a multiverse analysis corrected from multiple comparisons, in line with our global census analysis (supplementary appendix S3.2). To construct our multiverse of analysis, we alternatively operationalized the prevalence of unmarried men as either (i) the proportion of men who are single or (ii) the proportion of men who are single or divorced; alternatively measured those proportions within the 21 age bands described in supplementary appendix S3.2; and alternatively applied (i) a standard t-test, (ii–iv) a t-test weighted by the adult male population, the adult female population, or the total adult population of the county, or (v) a (non-parametric) Wilcoxon rank sum test. We also tested the effects of including or excluding the three counties outside of Utah with large Mormon populations in our group of polygynous counties. Additionally, we tested the effect of including or excluding the two counties of Utah with only 58 and 63 adult men enumerated, respectively, which would have otherwise been excluded by our sample criteria.

Allowing for all combinations of these different analytical decisions results in 3,360 model specifications (including our main specification). Then, given the social and demographic heterogeneity of the United States, even at this point in history, we tested the

association between the presence of Mormon polygyny and the prevalence of unmarried men by separately comparing the polygynous counties with the monogamous counties in the West, Midwest, Northeast, and South of the country, as those regions are currently defined (79). Then, after conducting the resulting 13,440 pairwise tests, we applied the Benjaminin-Hochberg method to correct for multiple comparisons (2).

Supplementary figures

Figure S1

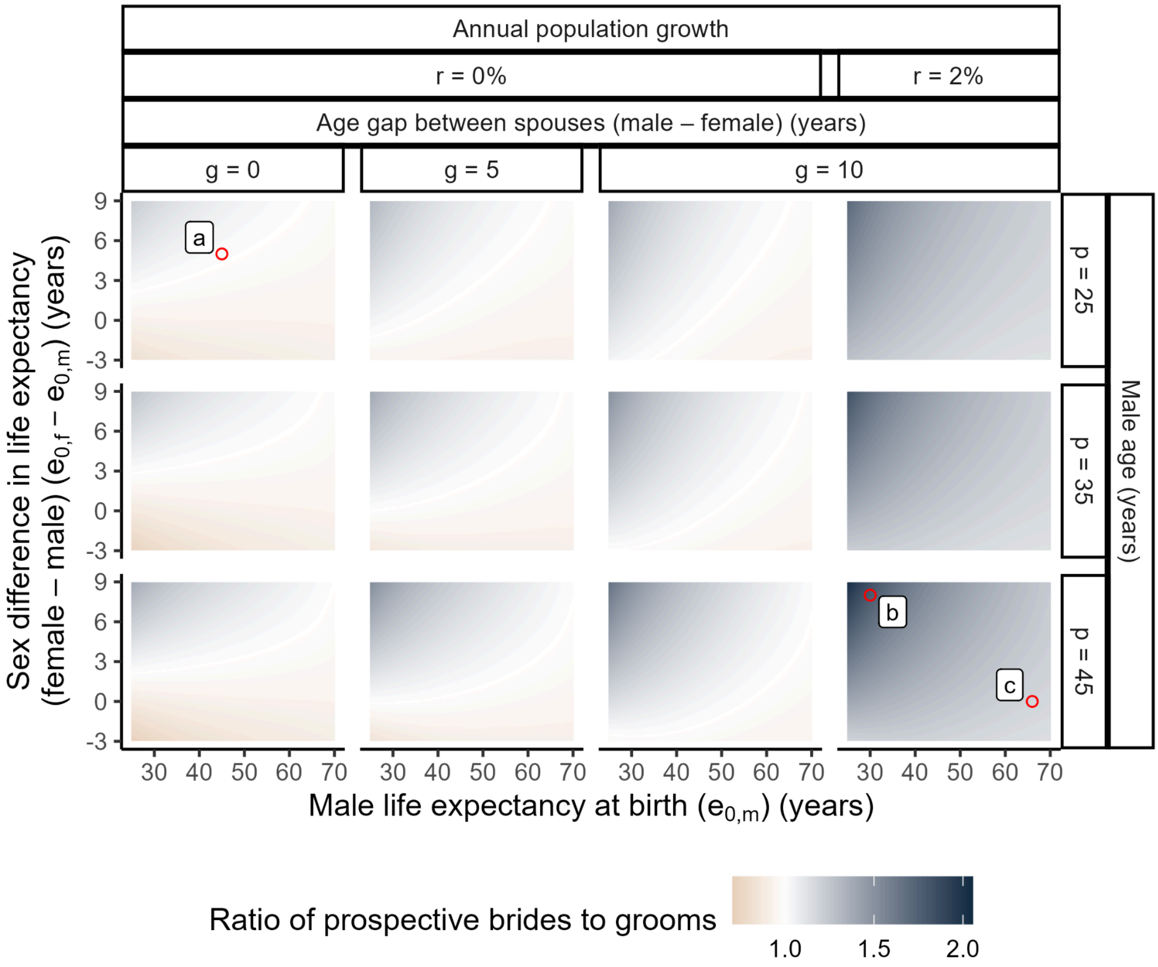

Figure S1. Modeled results of the sex ratios of prospective brides to grooms under a stable population regime that is closed to migration, as a function of female ($e_{0,f}$) and male ($e_{0,m}$) life expectancy, the annual population growth rate (r), male age (p), and the age gap between spouses (g). Three points (**a–c**) referenced in Section 1 are labeled on the plot.

Figure S2

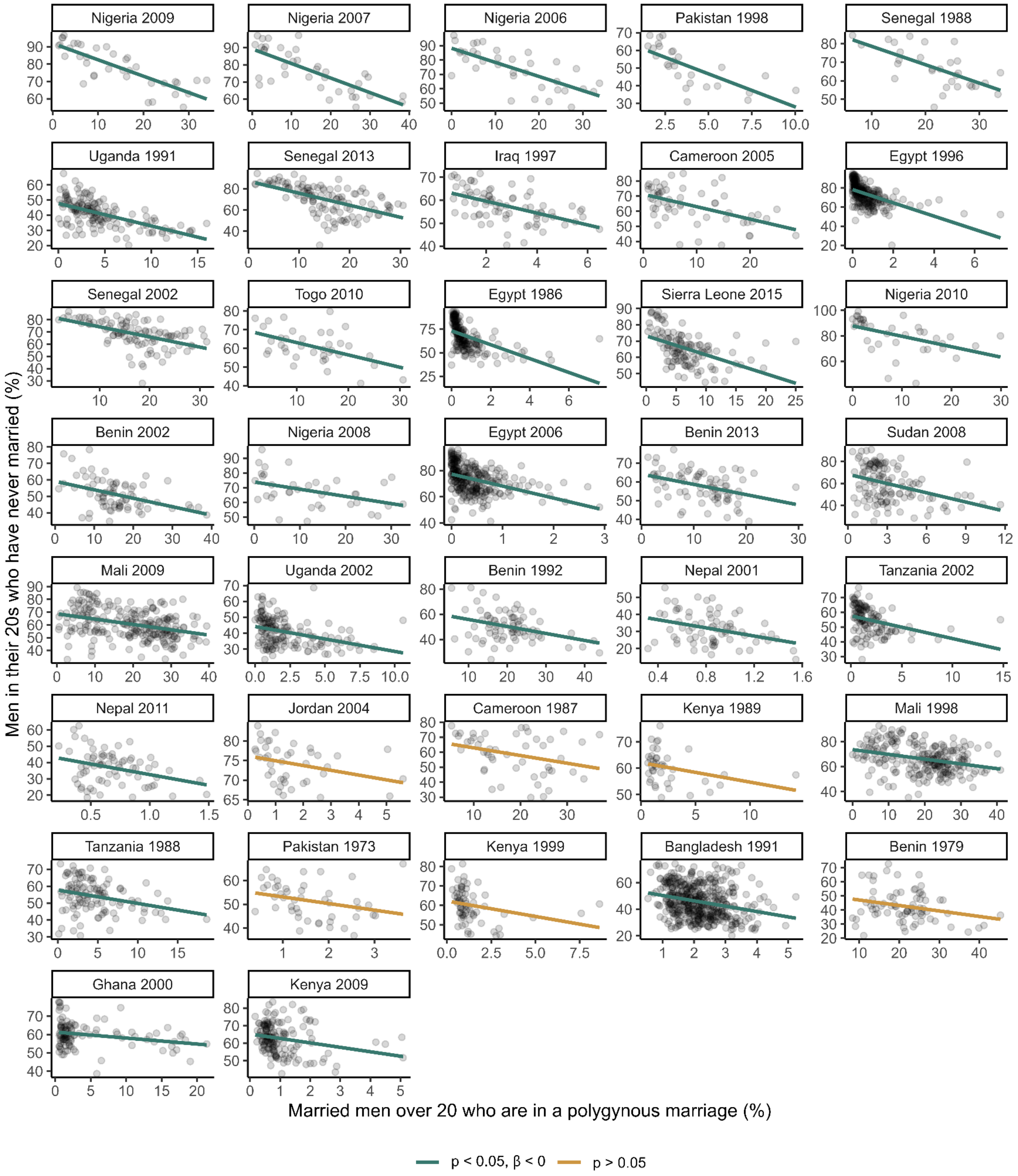

Figure S2. Sub-national associations between the prevalence of polygyny and the prevalence of unmarried men in the first 37 of the 74 censuses in the global analysis, sorted by the standardized coefficient of the association (from lowest to highest), under the main model model specification.

Figure S3

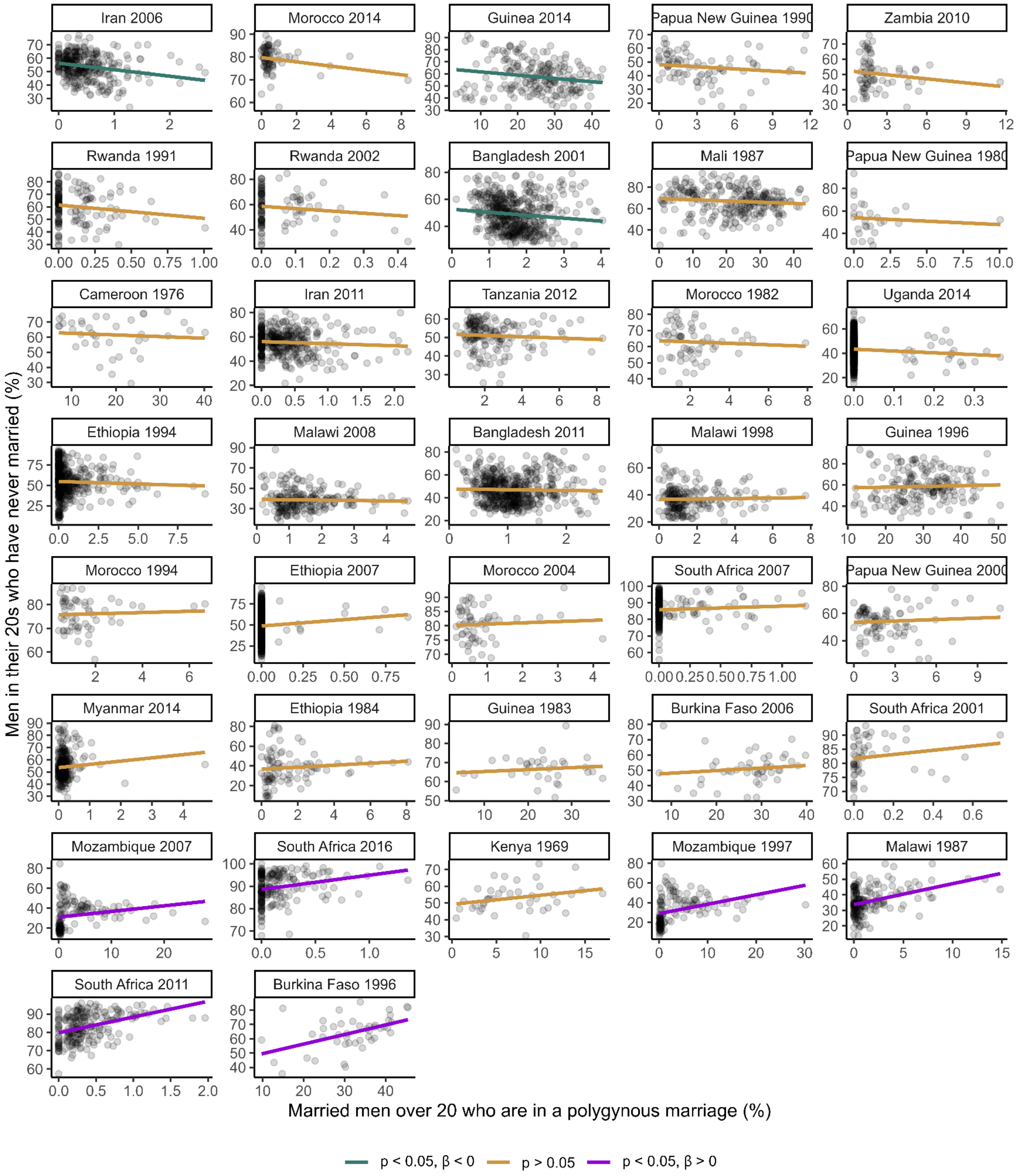

Figure S3. Sub-national associations between the prevalence of polygyny and the prevalence of unmarried men in the last 37 of the 74 censuses in the global analysis, sorted by the standardized coefficient of the association (from lowest to highest), under the main model model specification.

Figure S4

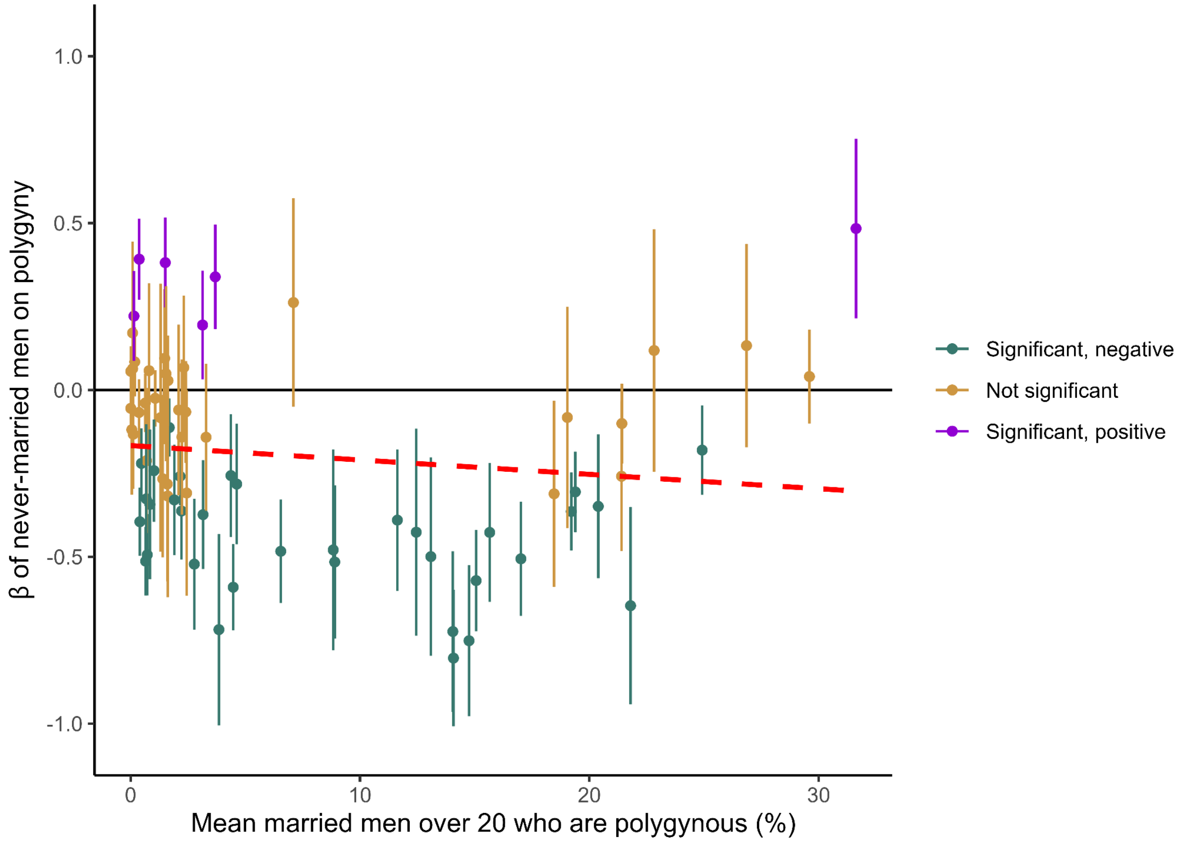

Figure S4. Within-census association between the local proportion of men over 20 in a polygynous marriage and the local proportion of men in their 20s who have never married, against the mean proportion of men over 20 in a polygynous marriage across the sub-national units of analysis for each census. The dashed red line is the ordinary least squares regression line, and the census-level coefficients are estimated under the main model specification.

Figure S5

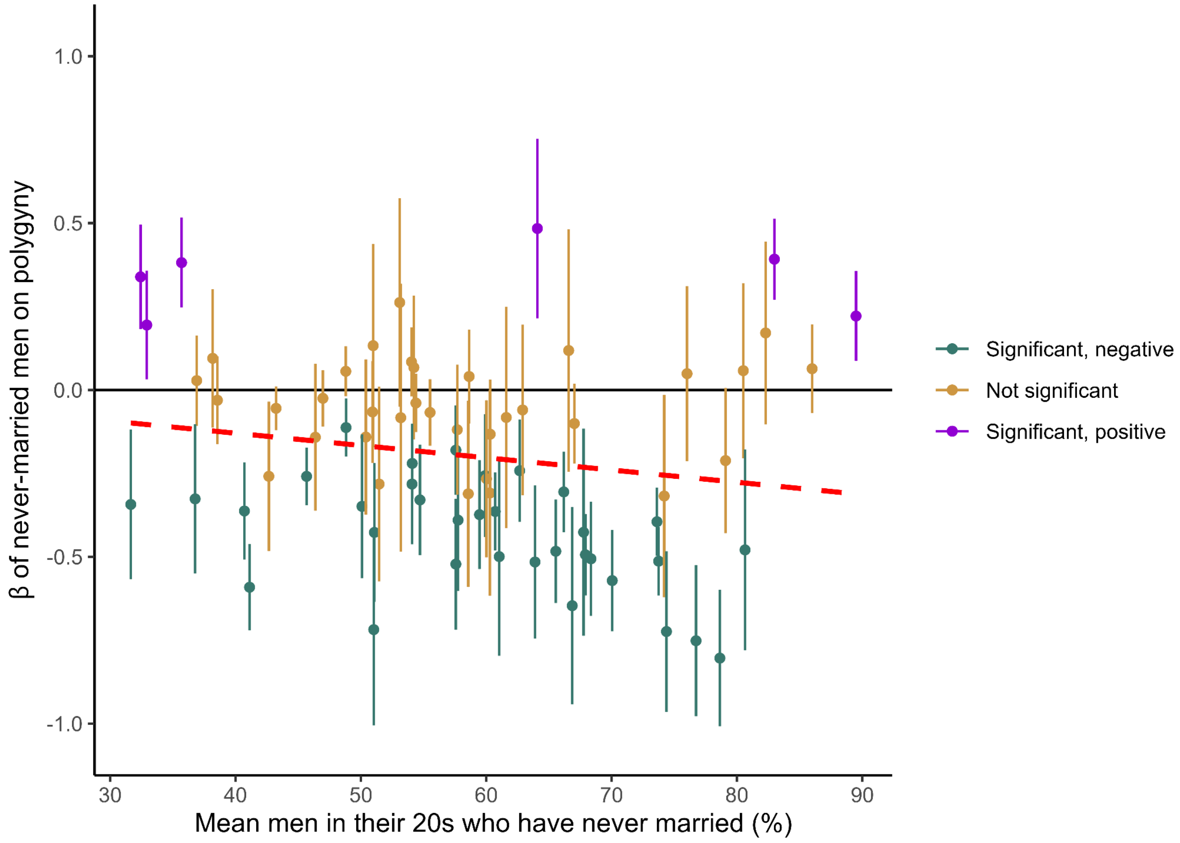

Figure S5. Within-census association between the local proportion of men over 20 in a polygynous marriage and the local proportion of men in their 20s who have never married, against the mean proportion of men in their 20s who have never married across the sub-national units of analysis for each census. The dashed red line is the ordinary least squares regression line, and the census-level coefficients are estimated under the main model specification.

Figure S6

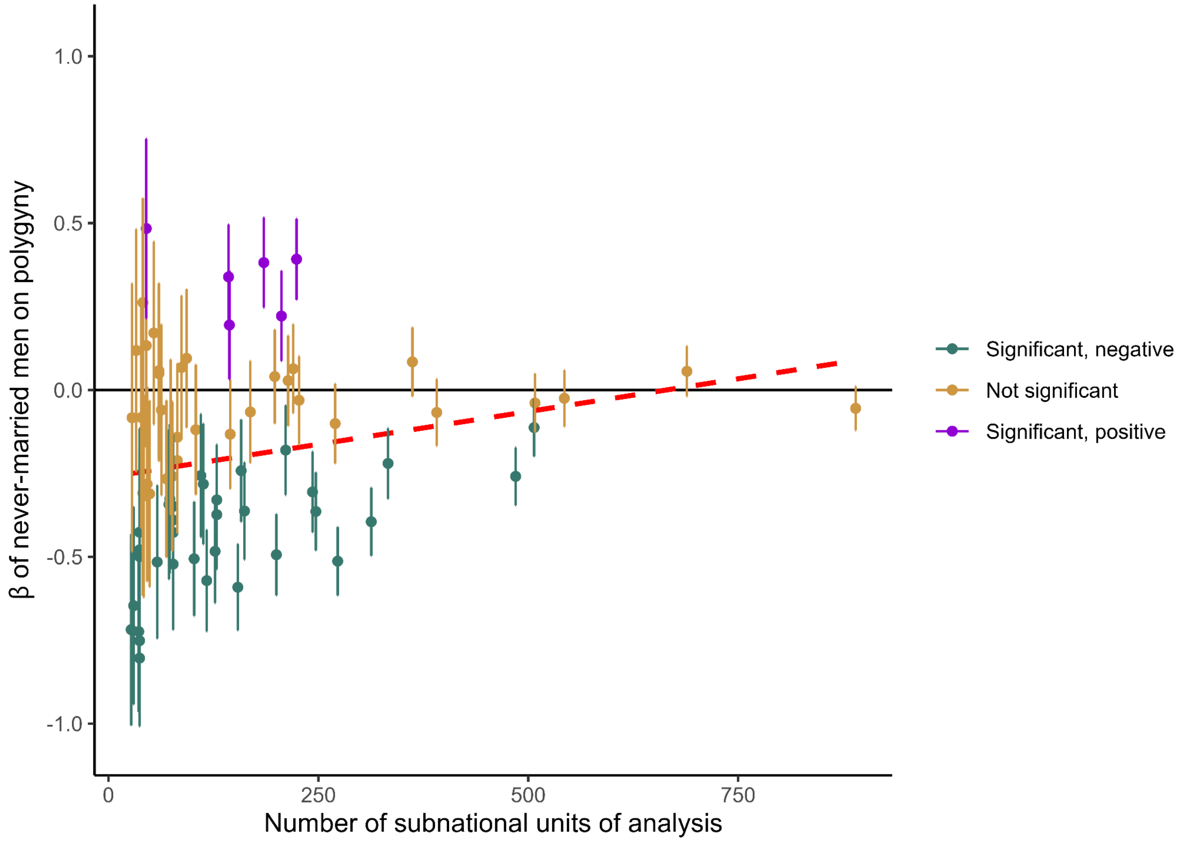

Figure S6. Within-census association between the local proportion of men over 20 in a polygynous marriage and the local proportion of men in their 20s who have never married, against the number of sub-national units of analysis for each census. The dashed red line is the ordinary least squares regression line, and the census-level coefficients are estimated under the main model specification.

Figure S7

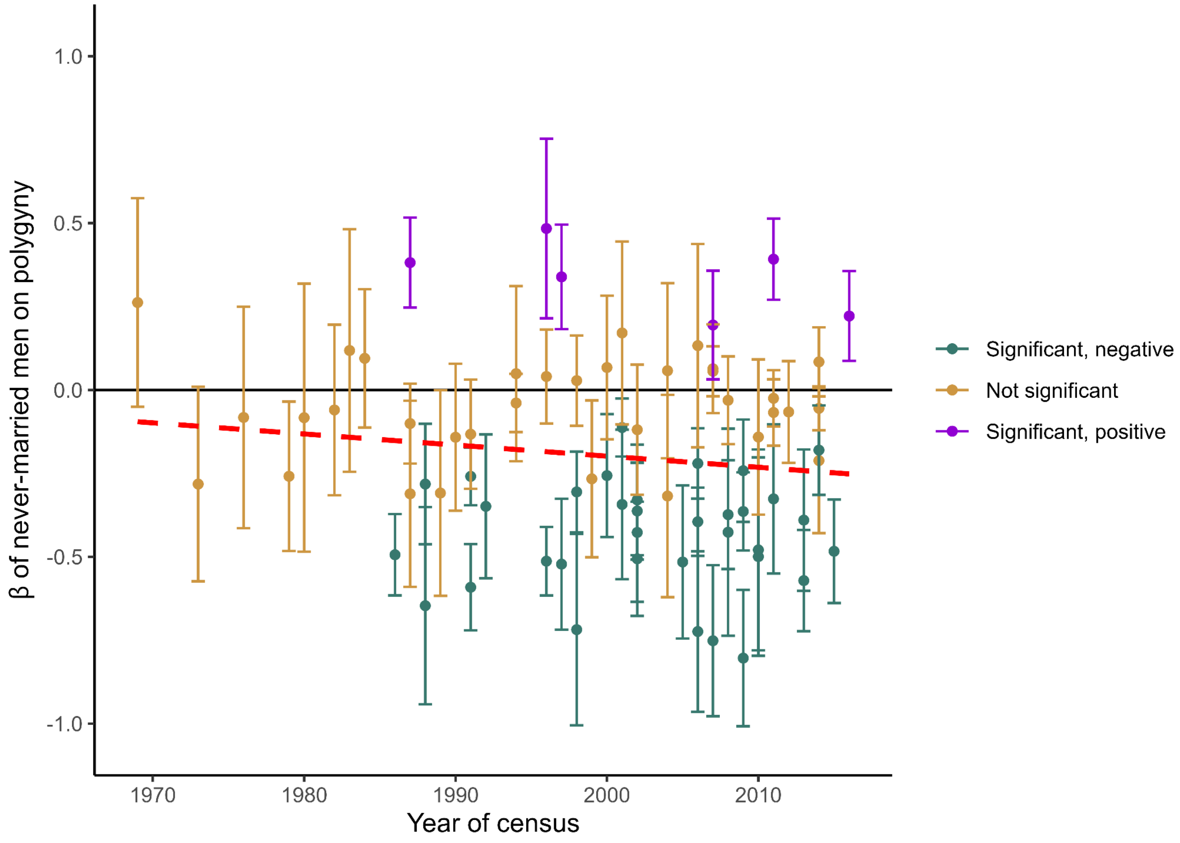

Figure S7. Within-census association between the local proportion of men over 20 in a polygynous marriage and the local proportion of men in their 20s who have never married, against the year of each census. The dashed red line is the ordinary least squares regression line, and the census-level coefficients are estimated under the main model specification.

Figure S8

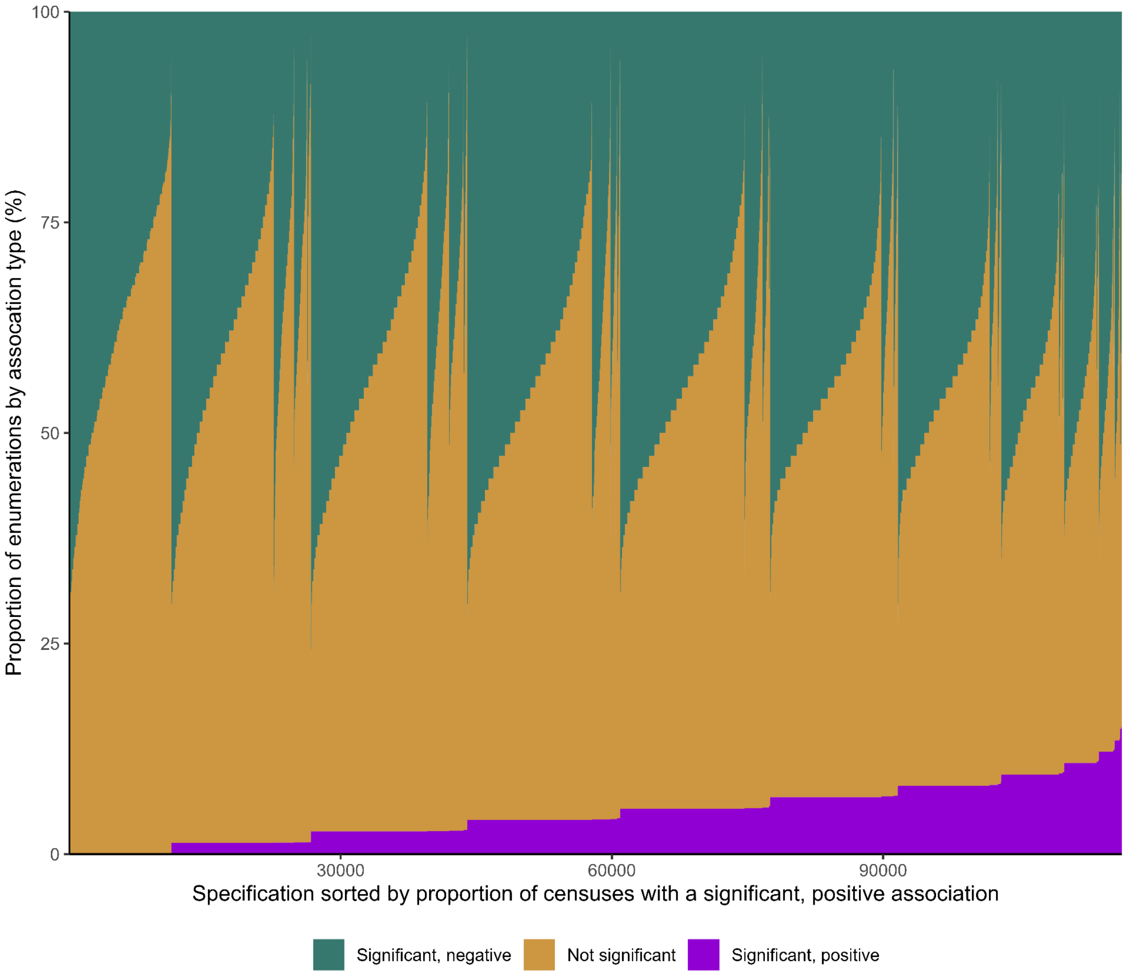

Figure S8. Summary of the associations between the prevalence of polygyny and the prevalence of unmarried men under 116,424 model specifications applied to the global sample.

Note: among all the specifications tested, the maximum proportion of censuses for which there is a significant positive association is only 18%, while the maximum proportion of significant negative associations found is 77%. Across all specifications, the median proportion of censuses with a significant positive association is only 4%, whereas the median proportion with a significant negative association is 43%.

Figure S9

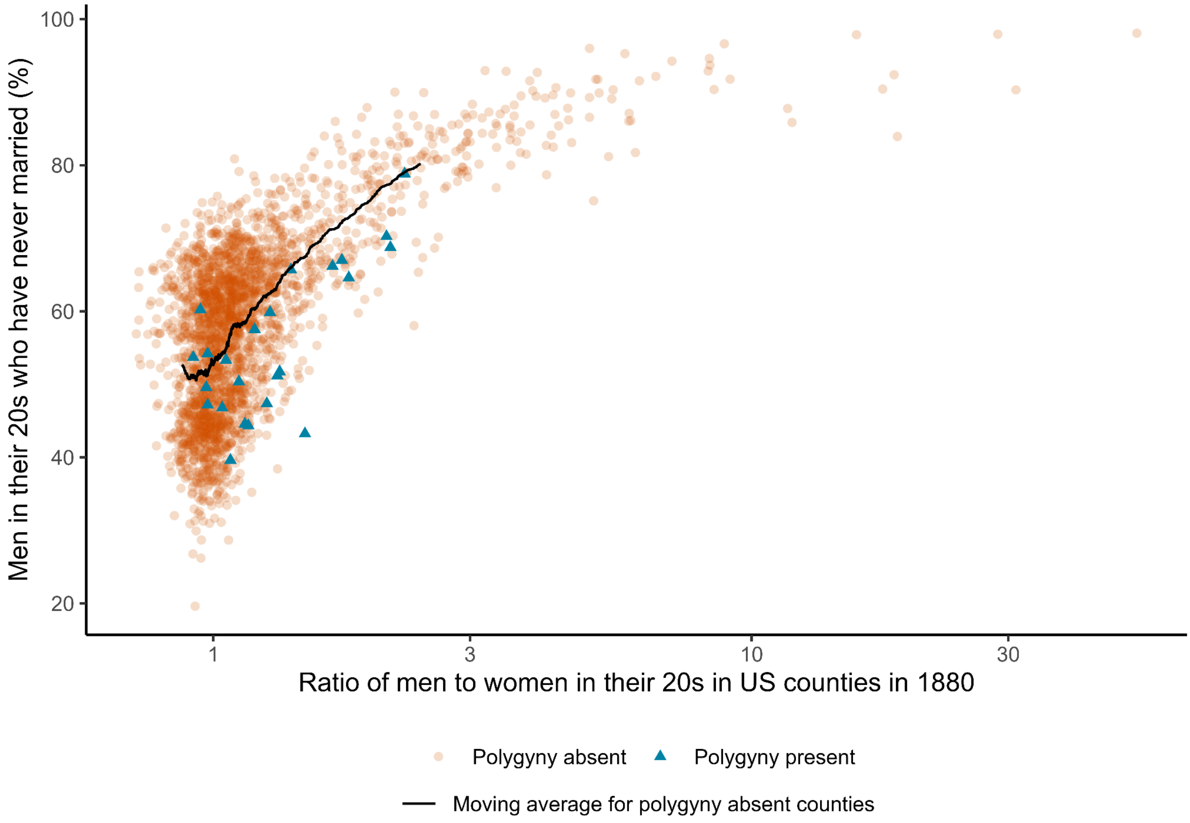

Figure S9. Proportion of men in their 20s who had never married across 2,475 US counties in 1880, disaggregated by the presence or absence of Mormon polygyny, against the ratio of men in their 20s to women in their 20s. Only counties with more than 100 men over age 20 enumerated in the census are shown. The black line is the moving average (period = 250) of the prevalence of unmarried men for the counties without Mormon polygyny, conditional on the population sex ratio.

Figure S10

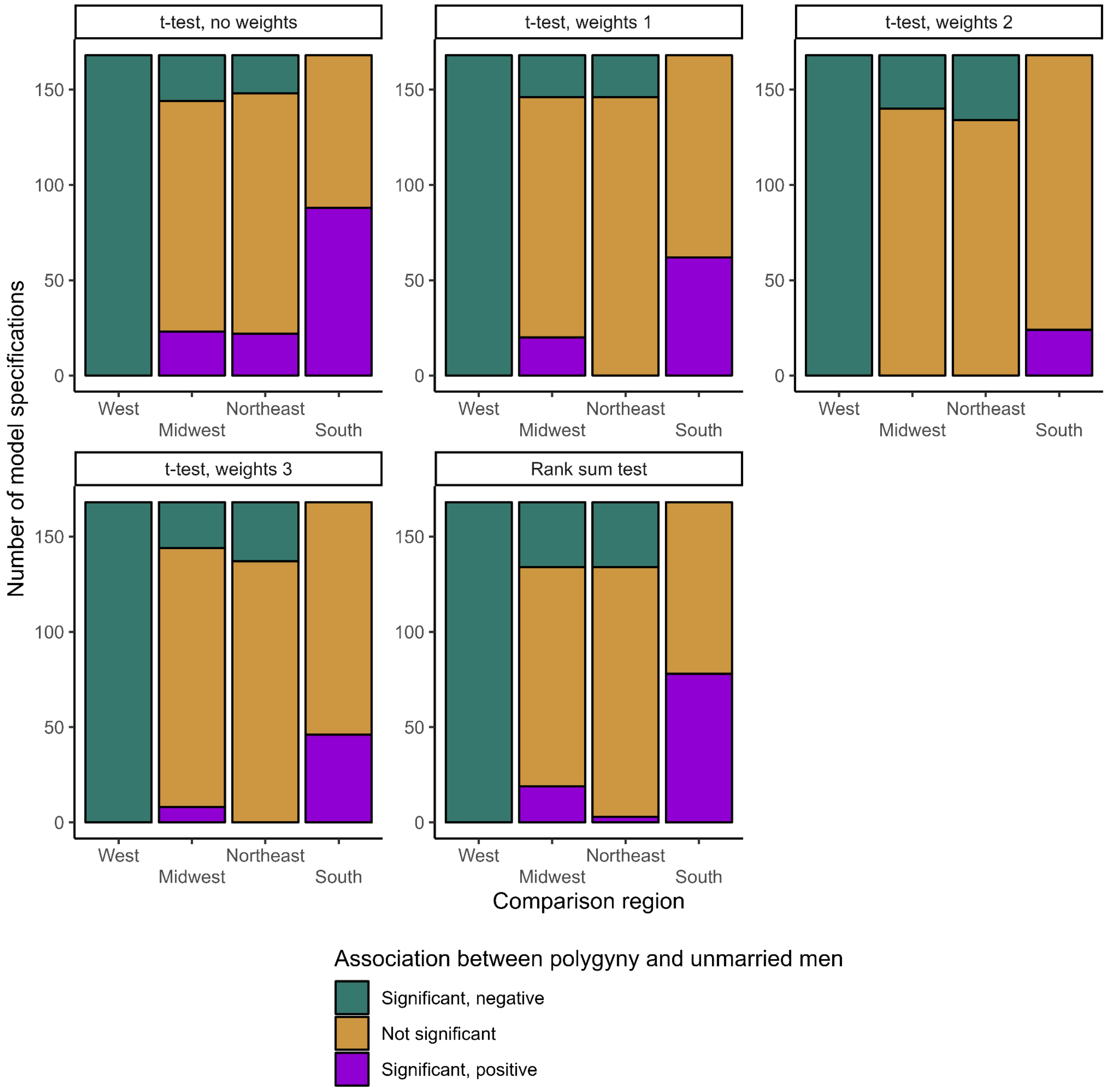

Figure S10. Summary of the associations between the presence of Mormon polygyny and the prevalence of unmarried men under 3,360 model specifications applied to the 1880 US sample, disaggregated by the statistical test used and by region. Pairwise comparisons are between counties of the West with Mormon polygyny and (other) counties in each region shown.

Supplementary tables

Table S1

Table S1. Summary table of the 85 sets of IPUMS International microdata with polygynous marriages recorded by the family interrelationship algorithm.

Census (country name and year conducted)	Sample of census available (%)	IPUMS variable for unit of analysis	All observed individuals age 20+ with known sex and marital status (n)	Individuals included in analysis, after exclusion criteria applied (%)	Total localities (n)	Localities included in analysis, after exclusion criteria applied (%)
Bangladesh 1991	10.00	GEO3_BD1991	4,915,812	100.0	485	100.0
Bangladesh 2001	10.00	GEO3_BD2001	6,374,609	100.0	507	100.0
Bangladesh 2011	5.00	GEO3_BD2011	4,067,183	100.0	543	100.0
Benin 1979	10.00	GEO2_BJ1979	143,443	100.0	76	100.0
Benin 1992	10.00	GEO2_BJ1992	206,794	100.0	77	100.0
Benin 2002	10.00	GEO2_BJ2002	295,076	100.0	77	100.0
Benin 2013	10.00	GEO2_BJ2013	435,861	100.0	77	100.0
Botswana 1981	10.00	GEO1_BW1981	42,879	0.0	23	0.0
Botswana 1991	10.00	GEO1_BW1991	59,963	0.0	22	0.0
Botswana 2001	10.00	GEO1_BW2001	85,463	0.0	22	0.0
Botswana 2011	10.00	GEO1_BW2011	114,160	0.0	22	0.0
Burkina Faso 1996	10.00	GEO2_BF1996	452,361	100.0	45	100.0
Burkina Faso 2006	10.00	GEO2_BF2006	589,878	100.0	45	100.0
Cameroon 1976	10.00	GEO2_CM1976	343,661	100.0	39	100.0
Cameroon 1987	10.00	GEO2_CM1987	398,219	100.0	49	100.0
Cameroon 2005	10.00	GEO2_CM2005	804,573	100.0	58	100.0
Egypt 1986	14.10	GEO2_EG1986	3,424,229	100.0	202	99.0
Egypt 1996	10.00	GEO2_EG1996	2,983,295	100.0	273	100.0
Egypt 2006	10.00	GEO2_EG2006	4,118,486	100.0	313	100.0
Ethiopia 1984	10.00	GEO2_ET1984	1,471,040	100.0	95	97.9
Ethiopia 1994	10.00	GEO3_ET1994	2,178,716	99.9	515	98.6
Ethiopia 2007	10.00	GEO3_ET2007	574,111	99.3	714	96.5
Ghana 2000	10.00	GEO2_GH2000	922,011	100.0	110	100.0
Guinea 1983	10.00	GEO1_GN1983	225,174	100.0	33	100.0
Guinea 1996	10.00	GEO2_GN1996	307,147	100.0	198	100.0
Guinea 2014	10.00	GEO2_GN2014	468,092	100.0	211	100.0
Iran 2006	2.00	GEO2_IR2006	789,652	100.0	333	100.0
Iran 2011	2.00	GEO2_IR2011	1,005,054	100.0	391	100.0
Iraq 1997	10.00	GEO2_IQ1997	861,543	100.0	77	100.0

Jordan 2004	10.00	GEO2_JO2004	259,067	100.0	42	100.0
Kenya 1969	6.00	GEO2_KE1969	303,565	100.0	41	100.0
Kenya 1989	5.00	GEO2_KE1989	434,275	100.0	41	100.0
Kenya 1999	5.00	GEO2_KE1999	627,521	100.0	69	100.0
Kenya 2009	10.00	GEO2_KE2009	1,772,143	100.0	158	100.0
Liberia 2008	10.00	GEO1_LR2008	164,335	0.0	15	0.0
Malawi 1987	10.00	GEO2_MW1987	345,810	100.0	185	100.0
Malawi 1998	10.00	GEO2_MW1998	450,317	100.0	214	100.0
Malawi 2008	10.00	GEO2_MW2008	578,344	100.0	227	100.0
Mali 1987	10.00	GEO3_ML1987	342,419	100.0	270	100.0
Mali 1998	10.00	GEO3_ML1998	424,087	100.0	243	100.0
Mali 2009	10.00	GEO3_ML2009	572,060	100.0	247	100.0
Morocco 1982	5.00	GEO2_MA1982	471,122	100.0	63	100.0
Morocco 1994	5.00	GEO2_MA1994	666,800	100.0	60	100.0
Morocco 2004	5.00	GEO2_MA2004	862,106	100.0	60	100.0
Morocco 2014	10.00	GEO2_MA2014	2,101,895	100.0	84	97.6
Mozambique 1997	10.00	GEO2_MZ1997	677,419	100.0	143	100.0
Mozambique 2007	10.00	GEO2_MZ2007	868,352	100.0	144	100.0
Myanmar 2014	10.00	GEO3_MM2014	3,130,280	100.0	362	100.0
Nepal 2001	9.00	GEO2_NP2001	1,044,106	100.0	72	100.0
Nepal 2011	12.00	GEO2_NP2011	1,743,300	100.0	73	100.0
Nigeria 2006	0.06	GEO1_NG2006	40,106	99.7	37	97.3
Nigeria 2007	0.06	GEO1_NG2007	39,723	100.0	37	100.0
Nigeria 2008	0.07	GEO1_NG2008	50,140	100.0	37	100.0
Nigeria 2009	0.05	GEO1_NG2009	37,116	100.0	37	100.0
Nigeria 2010	0.05	GEO1_NG2010	36,246	100.0	38	97.4
Pakistan 1973	2.00	GEO2_PK1973	687,559	100.0	46	100.0
Pakistan 1998	10.00	GEO2_PK1998	6,054,075	100.0	27	100.0
Palestine 1997	10.00	GEO1_PS1997	109,360	0.0	16	0.0
Palestine 2007	10.00	GEO1_PS2007	103,658	0.0	11	0.0
Palestine 2017	10.00	GEO1_PS2017	236,266	0.0	16	0.0
Papua New Guinea 1980	10.00	GEO2_PG1980	29,025	91.7	81	34.6
Papua New Guinea 1990	10.00	GEO2_PG1990	169,796	100.0	82	100.0
Papua New Guinea 2000	10.00	GEO2_PG2000	253,107	100.0	87	100.0
Rwanda 1991	10.00	GEO2_RW1991	301,044	100.0	145	100.0
Rwanda 2002	10.00	GEO2_RW2002	338,677	100.0	104	100.0
Senegal 1988	10.00	GEO2_SN1988	297,414	100.0	30	100.0
Senegal 2002	10.00	GEO3_SN2002	451,975	100.0	102	100.0
Senegal 2013	10.00	GEO3_SN2013	567,357	100.0	117	100.0

Sierra Leone 2015	10.00	GEO2_SL2015	331,760	100.0	127	100.0
South Africa 2001	10.00	GEO2_ZA2001	2,095,991	100.0	54	100.0
South Africa 2007	2.00	GEO3_ZA2007	547,854	100.0	220	100.0
South Africa 2011	8.60	GEO3_ZA2011	2,665,548	100.0	224	100.0
South Africa 2016	5.80	GEO3_ZA2016	2,042,230	100.0	206	100.0
South Sudan 2008	7.00	GEO1_SS2008	230,413	0.0	10	0.0
Sudan 2008	16.60	GEO2_SD2008	2,295,112	100.0	129	100.0
Tanzania 1988	10.00	GEO2_TZ1988	1,009,194	100.0	113	100.0
Tanzania 2002	10.00	GEO2_TZ2002	1,713,407	100.0	129	100.0
Tanzania 2012	10.00	GEO2_TZ2012	2,035,493	100.0	169	100.0
Togo 1960	10.00	GEO1_TG1960	6,214	0.0	7	0.0
Togo 1970	1.00	GEO2_TG1970	10,277	0.0	19	0.0
Togo 2010	10.00	GEO2_TG2010	276,944	100.0	37	100.0
Uganda 1991	10.00	GEO2_UG1991	651,408	100.0	154	100.0
Uganda 2002	10.00	GEO2_UG2002	1,019,447	100.0	162	100.0
Uganda 2014	10.00	GEO3_UG2014	1,472,512	100.0	890	100.0
Zambia 2010	10.00	GEO2_ZM2010	531,538	100.0	74	100.0

Table S2

Table S2. Summary table of the prevalence of polygyny and the prevalence of unmarried men in censuses in the 85 sets of IPUMS International microdata with polygynous marriages recorded by the family interrelationship algorithm.

Census (country name and year conducted)	Observed men age 20+ who are in a polygynous union (%)	Observed women age 20+ who are married to a polygynous man (%)	Observed men age 20+ who are single (%)	Observed men age 20+ who are single or divorced (%)
Bangladesh 1991	1.742	3.490	17.1	17.1
Bangladesh 2001	1.345	2.616	19.2	19.3
Bangladesh 2011	0.853	1.613	16.5	16.7
Benin 1979	15.394	26.876	43.4	45.8
Benin 1992	13.296	24.155	43.9	46.0
Benin 2002	9.769	17.729	46.6	48.4
Benin 2013	7.240	13.205	46.1	47.6
Botswana 1981	0.433	0.795	24.6	26.1
Botswana 1991	0.203	0.348	28.2	31.5
Botswana 2001	0.020	0.035	28.8	31.9
Botswana 2011	0.020	0.042	35.0	36.7
Burkina Faso 1996	19.534	39.092	19.8	23.0
Burkina Faso 2006	17.702	32.438	25.8	28.3
Cameroon 1976	12.928	25.785	25.5	27.3
Cameroon 1987	11.671	22.793	26.8	28.2
Cameroon 2005	5.204	10.345	11.9	15.9
Egypt 1986	0.390	0.809	20.7	24.3
Egypt 1996	0.422	0.858	21.3	24.4
Egypt 2006	0.257	0.532	23.1	23.6
Ethiopia 1984	1.224	2.108	26.6	26.6
Ethiopia 1994	0.413	0.761	26.7	27.3
Ethiopia 2007	0.005	0.009	28.2	33.9
Ghana 2000	2.296	4.615	28.1	28.9
Guinea 1983	15.470	30.233	26.6	28.2
Guinea 1996	19.494	35.648	29.3	29.8
Guinea 2014	15.284	27.885	21.5	21.9
Iran 2006	0.316	0.621	22.8	23.7
Iran 2011	0.198	0.378	30.0	30.8
Iraq 1997	1.395	2.676	33.2	33.6
Jordan 2004	0.704	1.473	24.5	26.4
Kenya 1969	3.127	8.642	28.5	30.1
Kenya 1989	1.596	2.924	29.3	31.3
Kenya 1999	0.853	1.534	29.2	31.4
Kenya 2009	0.649	1.188	34.6	38.8
Liberia 2008	1.041	2.096	14.3	17.9
Malawi 1987	1.028	1.818	15.7	19.1
Malawi 1998	1.273	2.355	17.1	19.8

Malawi 2008	1.140	2.157	26.8	27.5
Mali 1987	15.084	27.417	28.9	29.5
Mali 1998	13.010	24.706	27.1	27.6
Mali 2009	12.753	24.219	26.3	27.8
Morocco 1982	1.399	2.629	34.0	35.0
Morocco 1994	0.827	1.559	35.6	36.5
Morocco 2004	0.384	0.725	32.4	33.5
Morocco 2014	0.244	0.472	15.1	17.8
Mozambique 1997	1.862	3.099	15.7	18.7
Mozambique 2007	1.456	2.488	12.1	12.5
Myanmar 2014	0.106	0.179	13.2	13.5
Nepal 2001	0.695	1.306	24.6	27.7
Nepal 2011	0.538	0.915	25.5	28.0
Nigeria 2006	12.576	24.911	22.1	23.8
Nigeria 2007	11.641	22.670	27.2	29.3
Nigeria 2008	10.582	20.966	28.5	30.3
Nigeria 2009	10.844	21.430	20.0	21.3
Nigeria 2010	7.007	14.202	24.2	24.4
Pakistan 1973	0.884	2.058	32.3	34.7
Pakistan 1998	2.186	4.809	21.5	23.4
Palestine 1997	1.065	2.144	24.5	26.9
Palestine 2007	0.544	1.101	27.3	29.1
Palestine 2017	0.369	0.746	28.8	29.7
Papua New Guinea 1980	0.670	2.017	32.4	33.8
Papua New Guinea 1990	2.391	5.364	35.8	36.4
Papua New Guinea 2000	1.616	3.459	36.3	37.4
Rwanda 1991	0.087	0.138	36.0	38.7
Rwanda 2002	0.033	0.051	42.5	45.1
Senegal 1988	13.335	25.752	45.1	47.2
Senegal 2002	9.213	18.437	46.1	48.2
Senegal 2013	7.618	14.711	52.8	54.8
Sierra Leone 2015	3.511	6.646	29.0	30.3
South Africa 2001	0.034	0.058	26.7	27.9
South Africa 2007	0.032	0.054	29.6	32.9
South Africa 2011	0.149	0.224	19.4	22.3
South Africa 2016	0.056	0.094	28.7	31.3
South Sudan 2008	1.464	2.627	22.3	28.9
Sudan 2008	2.247	4.216	20.5	25.4
Tanzania 1988	3.386	6.161	20.5	24.8
Tanzania 2002	1.371	2.401	27.9	28.5
Tanzania 2012	1.715	3.064	26.3	26.7
Togo 1960	12.022	22.038	28.3	28.7
Togo 1970	21.061	35.855	24.2	28.4
Togo 2010	7.053	12.853	25.7	29.5
Uganda 1991	2.979	5.320	23.0	26.0

Uganda 2002	1.598	2.994	33.7	34.3
Uganda 2014	0.004	0.006	25.4	26.2
Zambia 2010	1.496	2.823	25.4	28.6

Supplementary references

1. J. Henrich, R. Boyd, P. J. Richerson, The puzzle of monogamous marriage. *Phil. Trans. R. Soc. B* **367**, 657–669 (2012).

2. Y. Benjamini, Y. Hochberg, Controlling the False Discovery Rate: A Practical and Powerful Approach to Multiple Testing. *Journal of the Royal Statistical Society: Series B (Methodological)* **57**, 289–300 (1995).

3. D. A. Savitz, G. A. Wellenius, Can Cross-Sectional Studies Contribute to Causal Inference? It Depends. *American Journal of Epidemiology* **192**, 514–516 (2023).

4. M. Ember, Warfare, Sex Ratio, and Polygyny. *Ethnology* **13**, 197 (1974).

5. L. L. Betzig, *Despotism and differential reproduction: a darwinian view of history* (Routledge, 2018).

6. K. S. Gleditsch, J. Wucherpfennig, S. Hug, K. G. Reigstad, Polygyny or Misogyny? Reexamining the "First Law of Intergroup Conflict." *The Journal of Politics* **73**, 265–270 (2011).

7. C. T. Ross, *et al.*, Greater wealth inequality, less polygyny: rethinking the polygyny threshold model. *J. R. Soc. Interface.* **15**, 20180035 (2018).

8. R. Schacht, K. L. Rauch, M. Borgerhoff Mulder, Too many men: the violence problem? *Trends in Ecology & Evolution* **29**, 214–222 (2014).

9. K. Ash, Does polygyny cause intergroup conflict? Re-examining Koos and Neupert-Wentz (2020). *Research & Politics* **9**, 205316802211349 (2022).

10. J. M. Rexer, The Brides of Boko Haram: Economic Shocks, Marriage Practices, and Insurgency in Nigeria. *The Economic Journal* **132**, 1927–1977 (2022).

11. J. Mellon, Rain, rain, go away: 194 potential exclusion-restriction violations for studies using weather as an instrumental variable. *American J Political Sci* ajps.12894 (2024). https://doi.org/10.1111/ajps.12894.

12. S. Dawkins, The problem of the missing dead. *Journal of Peace Research* **58**, 1098–1116 (2021).

13. T. Y. Coulibaly, S. Managi, Identifying the impact of rainfall variability on conflicts at the monthly level. *Sci Rep* **12**, 18162 (2022).

14. H. Sarsons, Rainfall and conflict: A cautionary tale. *Journal of Development Economics* **115**, 62–72 (2015).

15. N. Li, P. Gerland, Model Life Tables. *United Nations Population Division* (2012). Available at: https://www.un.org/development/desa/pd/data/model-life-tables [Accessed 16 March 2023].

16. E. Van de Walle, "Marriage in African Censuses and Inquires" in *The Demography of Tropical Africa*, W. Brass, *et al.*, Eds. (Princeton University Press, 1968), pp. 183–238.

17. T. Locoh, La nuptialité au Togo. Evolution entre 1961 et 1970. *Population* **31**, 379–398 (1976).

18. P. Antoine, “The Complexities of Nuptiality: From Early Female Union to Male Polygamy in Africa” in *Demography: Analysis and Synthesis*, G. Caselli, J. Vallin, G. Wunsch, Eds. (Academic Press, 2006), pp. 355–371.

19. H. Chojnacka, Polygyny and the Rate of Population Growth. *Population Studies* **34**, 91–107 (1980).

20. H. G. Jacoby, The Economics of Polygyny in Sub-Saharan Africa: Female Productivity and the Demand for Wives in Côte d’Ivoire. *Journal of Political Economy* **103**, 938–971 (1995).

21. A. Morse, N. Luke, Foetal loss and feminine sex ratios at birth in sub-Saharan Africa. *Population Studies* **75**, 239–254 (2021).

22. F. J. Beltrán Tapia, M. Szołtysek, ‘Missing girls’ in historical Europe: reopening the debate. *The History of the Family* **27**, 619–657 (2022).

23. A. Marino, Family, fertility, and sex ratios in the British Caribbean. *Population Studies* **24**, 159–172 (1970).

24. D. A. Momeni, Polygyny in Iran. *Journal of Marriage and Family* **37**, 453–456 (1975).

25. R. Lesthaeghe, G. Kaufmann, D. Meekers, “The Nuptiality Regimes in Sub-Saharan Africa” in *Reproduction and Social Organization in Sub-Saharan Africa*, R. J. Lesthaeghe, Ed. (University of California Press, 1989), pp. 238–337.

26. T. Hovestadt, O. Mitesser, H.-J. Poethke, Gender-Specific Emigration Decisions Sensitive to Local Male and Female Density. *The American Naturalist* **184**, 38–51 (2014).

27. R. Kashyap, A. Esteve, J. García-Román, Potential (Mis)match? Marriage Markets Amidst Sociodemographic Change in India, 2005–2050. *Demography* **52**, 183–208 (2015).

28. M. Rahaman, A. Roy, N. Kapasia, P. Chouhan, Spousal violence in India: does risk of spousal violence higher among polygynous unions? *Cogent Social Sciences* **8**, 2103945 (2022).

29. M. Ní Bhrolcháin, Flexibility in the Marriage Market. *Population: An English Selection* **13**, 9–47 (2001).

30. T. Bergstrom, D. Lam, “The Effects of Cohort Size on Marriage Markets in Twentieth Century Sweden” (Center for Research on Economic and Social Theory, 1989).

31. A. Siow, How does the marriage market clear? An empirical framework. *Canadian J of Economics* **41**, 1121–1155 (2008).

32. R. Abramitzky, A. Delavande, L. Vasconcelos, Marrying Up: The Role of Sex Ratio in Assortative Matching. *American Economic Journal: Applied Economics* **3**, 124–157 (2011).

33. A. J. Gage-Brandon, The Polygyny-Divorce Relationship: A Case Study of Nigeria. *Journal of Marriage and Family* **54**, 285–292 (1992).

34. G. Reniers, Divorce and Remarriage in Rural Malawi. *DemRes* **Special 1**, 175–206

(2003).

35. P. Antoine, “Event-History Analysis of Nuptiality: An Application to Africa” in *Demography: Analysis and Synthesis*, G. Caselli, J. Vallin, G. Wunsch, Eds. (Academic Press, 2006), pp. 339–353.

36. S. Chae, V. Agadjanian, The Transformation of Polygyny in Sub-Saharan Africa. *Population & Development Rev* **48**, 1125–1162 (2022).

37. J. C. Riley, Estimates of Regional and Global Life Expectancy, 1800-2001. *Population & Development Review* **31**, 537–543 (2005).

38. J. Bor, *et al.*, Mass HIV Treatment and Sex Disparities in Life Expectancy: Demographic Surveillance in Rural South Africa. *PLoS Med* **12**, e1001905 (2015).

39. V. H. Chisumpa, C. O. Odimegwu, N. Saikia, Adult mortality in sub-Saharan Africa: cross-sectional study of causes of death in Zambia. *Tropical Med Int Health* **24**, 1208–1220 (2019).

40. M. Garenne, Sex Ratios at Birth in African Populations: A Review of Survey Data. *Human Biology* **74**, 889–900 (2002).

41. M. Luy, Causes of Male Excess Mortality: Insights from Cloistered Populations. *Population & Development Rev* **29**, 647–676 (2003).

42. G. L. Drevenstedt, E. M. Crimmins, S. Vasunilashorn, C. E. Finch, The rise and fall of excess male infant mortality. *Proc. Natl. Acad. Sci. U.S.A.* **105**, 5016–5021 (2008).

43. A. Ezeh, F. Kissling, P. Singer, Why sub-Saharan Africa might exceed its projected population size by 2100. *The Lancet* **396**, 1131–1133 (2020).

44. N. Goldman, A. R. Pebley, “The Demography of Polygyny in Sub-Saharan Africa” in *Reproduction and Social Organization in Sub-Saharan Africa*, R. Lesthaeghe, Ed. (University of California Press, 1989), pp. 212–237.

45. M. Tertilt, Polygyny, Fertility, and Savings. *Journal of Political Economy* **113**, 1341–1371 (2005).

46. M. A. Gibson, R. Mace, Polygyny, Reproductive Success and Child Health in Rural Ethiopia: Why Marry a Married Man? *J. Biosoc. Sci.* **39**, 287–300 (2007).

47. M. Boltz, I. Chort, The Risk of Polygamy and Wives’ Saving Behavior. *The World Bank Economic Review* **33**, 209–230 (2019).

48. M. K. Whyte, Cross-Cultural Codes Dealing with the Relative Status of Women. *Ethnology* **17**, 211–237 (1978).

49. G. J. Broude, S. J. Greene, Cross-Cultural Codes on Husband-Wife Relationships. *Ethnology* **22**, 263–280 (1983).

50. S. G. Frayser, *Varieties of sexual experience: an anthropological perspective on human sexuality* (HRAF Press, 1985).

51. P. McDonald, “Social organization and nuptiality in developing societies” in *Reproductive Change in Developing Countries*, J. Cleland, J. Hobcraft, Eds. (Oxford University Press, 1985), pp. 64–86.

52. A. Menashe-Oren, G. Stecklov, Rural/Urban Population Age and Sex Composition in sub-Saharan Africa 1980–2015. *Population and Development Review* **44**, 7–35 (2018).

53. A. Menashe-Oren, G. Stecklov, Age-specific sex ratios: Examining rural–urban variation within low- and middle-income countries. *Population Studies* **77**, 539–558 (2023).

54. B. Whitehouse, *Enduring polygamy: plural marriage and social change in an African metropolis* (Rutgers University, 2023).

55. Minnesota Population Center, Integrated Public Use Microdata Series, International: Version 7.3. Minneapolis, MN: IPUMS. https://doi.org/10.18128/D020.V7.3. Deposited 2020.

56. E. Smith-Greenaway, J. Trinitapoli, Polygynous Contexts, Family Structure, and Infant Mortality in Sub-Saharan Africa. *Demography* **51**, 341–366 (2014).

57. E. Coast, S. Randall, V. Golaz, B. Gnoumou, Problematic polygamy: implications of changing typologies and definitions of polygamy in (2011).

58. L. Holy, *Anthropological perspectives on kinship* (Pluto Press, 1996).

59. Y. Benjamini, D. Yekutieli, The control of the false discovery rate in multiple testing under dependency. *Ann. Statist.* **29**, 1165–1188 (2001).

60. T. G. Conley, M. Kelly, The standard errors of persistence. *Journal of International Economics* **153**, 104027 (2025).

61. M. Sobek, S. Kennedy, “The Development of Family Interrelationship Variables for International Census Data” (University of Minnesota, 2009).

62. J. A. Moorad, D. E. L. Promislow, K. R. Smith, M. J. Wade, Mating system change reduces the strength of sexual selection in an American frontier population of the 19th century. *Evolution and Human Behavior* **32**, 147–155 (2011).

63. C. R. Von Rueden, A. V. Jaeggi, Men’s status and reproductive success in 33 nonindustrial societies: Effects of subsistence, marriage system, and reproductive strategy. *Proc. Natl. Acad. Sci. U.S.A.* **113**, 10824–10829 (2016).

64. S. F. Faux, H. L. Miller, Evolutionary speculations on the oligarchic development of Mormon polygyny. *Ethology and Sociobiology* **5**, 15–31 (1984).

65. W. Jankowiak, Co-Wives, Husband, and the Mormon Polygynous Family. *Ethnology* **47**, 163–180 (2008).

66. R. Dorman, The Creation and Destruction of the 1890 Federal Census. *The American Archivist* **71**, 350–383 (2008).

67. S. Ruggles, R. R. Menard, A Public Use Sample of the 1880 U.S. Census of Population. *Historical Methods: A Journal of Quantitative and Interdisciplinary History* **23**, 104–115 (1990).

68. L. Logue, A Time of Marriage: Monogamy and Polygamy in a Utah Town. *Journal of Mormon History* **11**, 3–26 (1984).

69. K. M. Daynes, Striving to Live the Principle in Utah’s First Temple City: A Snapshot of Polygamy in St. George, Utah, in June 1880. *BYU Studies Quarterly* **51**, 69–95 (2012).

70. L. M. Logue, *A sermon in the desert: belief and behavior in early St. George, Utah* (University of Illinois Press, 1988).

71. L. B. Bennion, The Incidence of Mormon Polygamy in 1880: “Dixie” versus Davis Stake. *Journal of Mormon History* **11**, 27–42 (1984).

72. M. Cornwall, C. Courtright, L. Van Beek, How Common the Principle? Women as Plural Wives in 1860. *Dialogue: A Journal of Mormon Thought* **26**, 139–153 (1993).

73. L. C. “Ben” Bennion, Mapping the Extent of Plural Marriage in St. George, 1861-1880. *BYU Studies Quarterly* **51**, 27–68 (2012).

74. K. M. Daynes, Single Men in a Polygamous Society: Male Marriage Patterns in Manti, Utah. *Journal of Mormon History* **24**, 89–111 (1998).

75. S. A. Smith, “The Wasp in the Beehive: Non-Mormon Presence in 1880s Utah,” Pennsylvania State University, University Park, PA. (2008).

76. R. Sherlock, Mormon Migration and Settlement after 1875. *Journal of Mormon History* **2**, 53–68 (1975).

77. L. G. Coates, P. G. Boag, R. L. Hatzenbuehler, M. R. Swanson, The Mormon Settlement of Southeastern Idaho, 1845-1900. *Journal of Mormon History* **20**, 45–62 (1994).

78. M. E. Miller, St. Johns’s Saints: Interethnic Conflict in Northeastern Arizona, 1880-85. *Journal of Mormon History* **23**, 66–99 (1997).

79. US Census Bureau, Geographic Levels. *Census.gov* (2021). Available at: https://www.census.gov/programs-surveys/economic-census/guidance-geographies/levels.html [Accessed 19 August 2023].
